# ﻿Taxonomic revision of genus *Verbascum* (Scrophulariaceae) in the Arabian Peninsula

**DOI:** 10.3897/phytokeys.268.168267

**Published:** 2025-12-29

**Authors:** Ali Mohammed Alzahrani, Joana Magos Brehm, Shahina A. Ghazanfar, Nigel Maxted

**Affiliations:** 1 Department of Biology, Faculty of Science, Al-Baha University, Al-Baha, Saudi Arabia Al-Baha University Al-Baha Saudi Arabia; 2 School of Biosciences, University of Birmingham, Edgbaston, Birmingham, B15 2TT, UK University of Birmingham Edgbaston United Kingdom; 3 Royal Botanic Gardens, Kew, Richmond, Surrey, TW9 3AE, UK Royal Botanic Gardens Richmond United Kingdom

**Keywords:** Arabian Peninsula, new combination, new record, new species, *
Rhabdotosperma
*, *
Verbascum
*

## Abstract

The species of the genus *Verbascum* L. in the Arabian Peninsula are revised. Seventeen species are recognized, a key to the species is provided, and all names are typified. Detailed morphological descriptions, distribution maps, habitat information, and field images are presented for each species. *Verbascum sarawaticum* A. Alzahrani is newly described, and *Verbascum eremobium* Murb. is newly confirmed for the flora of the Arabian Peninsula. Seven new synonyms are established in this study. Accordingly, *Verbascum sheilae* Hemaid is treated as a variety under *V. deserticola* (Murb.) Huber-Morath (as var. sheilae); *Verbascum tabukum* Hemaid is placed in synonymy with *V. eremobium* Murb.; *Verbascum luntii* Baker is placed in synonymy with *V. longibracteatum* Defl.; *Verbascum hema-figranum* Hemaid is placed in synonymy with *V. medinecum* Hemaid; *Verbascum abyadicum* Hemaid is placed in synonymy with *V. shiqricum* Hemaid; and both *Verbascum chaudharyanum* Hemaid and *Verbascum asiricum* Hemaid are treated as varieties under *V. yemense* Defl. (as var. yemense and var. asiricum, respectively). Furthermore, the previously segregated genus *Rhabdotosperma* Hartl is here reduced to synonymy under *Verbascum* L., and the species formerly known as *Rhabdotosperma saudiarabicum* A. Alzahrani is formally recombined as *Verbascum saudiarabicum* (A. Alzahrani) A. Alzahrani.

## ﻿Introduction

*Verbascum* L. is the largest genus in the figwort family (Scrophulariaceae), with a long and complex taxonomic history due to the extreme similarity and hybridization among its taxa (Huber-Morath 1978). Linnaeus, in *Species Plantarum* (1753), considered the genus to be divided into *Celsia* L. and *Verbascum* based on the number of stamens, with the former having four and the latter five. Schrader (1813), the author of the first monograph on *Verbascum*, recognized sixty species based on characters such as the presence or absence of decurrent cauline leaves, in which the leaf base extends downward along the stem, and the number of flowers in the axil of each bract. Later, Berchtold and Pfund (1840) edited a second monograph on *Verbascum*, separating species based on whether the flowers were grouped, consisting of two groups of species with or without decurrent leaves, or solitary, consisting of two groups of species with one or multiple types of stamens. In addition, Bentham (1846) divided *Celsia* and *Verbascum* into two sections based on stamen type: sect. Thapsus Benth., characterized by anthers with connectives that extend downward onto the filament and appear slightly elongated, and sect. Lychnitis Benth., characterized by reniform (kidney-shaped) anthers, and applied this classification to both genera. Following this study, Franchet (1875) and Boissier (1879) treated *Verbascum* species within two sections based on Bentham’s classification and taxonomic distinctions.

Furthermore, Murbeck published detailed monographs on the genera *Celsia* (Murbeck, 1925) and *Verbascum* (Murbeck, 1933). The first (Murbeck 1925) divided the genus *Celsia* into two sections, Bothrospermae Murb. and Aulacospermae Murb., based on seed morphology, and the former section was further subdivided into subsections *Nefflea* Benth., in which all anthers are reniform, and *Arcturus* Benth., in which two anterior anthers are decurrent. In the second monograph on *Verbascum* (Murbeck, 1933), species were divided into the sections Bothrospermae Murb. and Aulacospermae Murb. based on seed morphology, and the former was further split into two subsections, *Fasciculata* Murb., characterized by clustered flowers, and *Singuliflora* Murb., characterized by solitary flowers. Later, Ferguson (1971) and Huber-Morath (1973) included *Celsia* and *Staurophragma* Fisch. & Mey. within *Verbascum* due to morphological similarity and the difficulty of distinguishing their species, as already observed in some critical plant genus groups (Perrino et al. 2022; Ben Mahmoud et al. 2024). Huber-Morath (1978) revised *Verbascum* species in Turkey and grouped the species of sect. Bothrospermae into 13 artificial groups according to the number of stamens, the type of hairs in the indumentum, and the number of flowers per bract.

*Rhabdotosperma* Hartl, which had previously been split between *Celsia* and Verbascum sect. Aulacospermae byMurbeck (1925, 1933), was separated as a distinct genus from *Verbascum* based on seed morphology by Hartl (1977). Use of *Rhabdotosperma* was subsequently followed by Lobin and Porembski (1994), Fischer (2004, 2006), Ghazanfar et al. (2008), and Alzahrani et al. (2022). Some authors, however, considered *Rhabdotosperma* a synonym of *Verbascum* due to their morphological similarities (Huber-Morath 1984; Wood 1997; Collenette 1999; Chaudhary 2001). Recent phylogenetic studies of *Verbascum* (Remal 2014; Ghahremaninejad et al. 2015; Sotoodeh 2015; Dong et al. 2022; Alzahrani et al. 2024) are not fully consistent with the traditional classifications of Murbeck (1925, 1933) and Huber-Morath (1973) but confirmed the monophyly of the genus and supported the inclusion of *Celsia*, *Staurophragma*, and *Rhabdotosperma* within *Verbascum*, as applied by Ferguson (1971) and Huber-Morath (1973, 1984). Although these molecular analyses recover several well-supported clades, they do not fully resolve all sister-species relationships, particularly among Arabian taxa relevant to this revision. Therefore, references to closely related species in this work refer to phylogenetically supported clades when available or to morphologically similar taxa where molecular resolution is limited.

Recent phylogenetic research based on DNA barcoding has also provided the first molecular framework specifically focused on Arabian *Verbascum* (Alzahrani et al. 2024). Using one nuclear marker, internal transcribed spacer (ITS), and three chloroplast regions, rbcL, matK, and trnL, analyzed under Maximum Parsimony and Bayesian Inference, this study recovered several well-supported clades that align closely with morphological species concepts while clarifying relationships among historically problematic taxa. Although the individual markers varied in resolution, the combined dataset consistently identified distinct lineages for several Arabian species. These molecular results therefore offer independent support for the taxonomic conclusions adopted in the present revision. Accordingly, statements throughout the species accounts referring to phylogenetic analyses specifically refer to the DNA barcoding evidence presented by Alzahrani et al. (2024), which complements the morphological assessments used here.

Current taxonomists rely on Murbeck’s (1933) classification in terms of seed morphology and divide species between Bothrospermae, characterized by transversely elongated seeds, and Aulacospermae, characterized by longitudinally furrowed seeds. In bothrospermous seeds, the alveolated endosperm follows the *Scrophularia*-type of alveolation, in which the endothelial cells divide unequally, producing a single enlarged cell, the bothroplast, and numerous smaller cells (Hartl 1959). The bothroplast protrudes into the endosperm and forms rounded alveoli; when these alveoli fuse, they give rise to the longitudinal furrows characteristic of aulacospermous seeds. This developmental process underlies the morphological distinction between bothrospermous and aulacospermous seed types in *Verbascum*. Thus, all Verbascum species belong to sect. Bothrosperma and comprise about 350 species distributed worldwide, mostly in western Asia, whereas Aulacospermae contains eight species found in tropical Africa and the Arabian Peninsula (Murbeck 1933; Huber-Morath 1973; Hartl 1977; Fischer 2004; Heywood et al. 2007; Christenhusz et al. 2017; Alzahrani et al. 2022).

The Middle East, Turkey, and Iran represent the center of diversity for *Verbascum*, where about 287 species have been recorded (Murbeck 1933, 1939; Huber-Morath 1978; Sharifnia 2007; Ranjbar and Nouri 2015; Sotoodeh 2015) and where the number of described species is increasing rapidly (Karavelioğulları et al. 2004; Parolly and Tan 2007; Sharifnia and Assadi 2007; Parolly and Eren 2008; Bani et al. 2010; Sotoodeh et al. 2015; Çingay et al. 2018; Ulukuş et al. 2020; Firat 2022; Sotoodeh et al. 2022).

Within the Arabian Peninsula, Deflers (1889) described the first two species of *Verbascum* from Yemen in *Voyage au Yemen*. Since then, accounts, checklists, and new species of *Verbascum* have been reported from other countries in the region (Baker 1894; Deflers 1896; Blatter 1921; Murbeck 1925, 1933; Migahid 1974; Huber-Morath 1984; Collenette 1985, 1998, 1999; Western 1989; Wood 1997; Al-Hemaid 2001; Chaudhary 2001; Jongbloed et al. 2003; Ghazanfar 1992, 2015; Alzahrani et al. 2022). Nevertheless, a comprehensive taxonomic revision of this genus in the Arabian Peninsula is timely given the widespread nomenclatural confusion and the absence of a useful key to *Verbascum* species. Therefore, this study provides the first detailed revision of *Verbascum* in the Arabian Peninsula.

## ﻿Materials and methods

*Verbascum* specimens and digital images were examined from the following herbaria: BM, E, K, KSU, MUZ, OBG, ON, and RIY, as well as through the JSTOR Global Plants platform (Thiers, continuously updated). All available specimens from the Arabian Peninsula were studied and cited unless otherwise indicated. The examination included assessment of indumentum type, leaf and floral morphology, measurement of diagnostic characters, and comparison with protologues and relevant taxonomic literature. Fieldwork was conducted in Saudi Arabia and Oman between 2019 and 2021, during which fresh specimens were collected, photographed, and compared directly with herbarium material to support the taxonomic conclusions presented here. The distribution map was generated using QGIS (2022) software version 3.22.

## ﻿Results

### ﻿Morphological characters

The following characteristics are useful for identifying and delimiting *Verbascum* species on the Arabian Peninsula.

#### ﻿Habit

Annual, biennial, or perennial herbs to small shrubs, ranging from 30 to 250 cm in height.

#### ﻿Stems

Stems are usually erect and terete but can sometimes be terete to angular (e.g., *V. bottae*, Fig. 3A). Branching can be a useful character, with stems being simple (e.g., *V. saudiarabicum*), branched from above (e.g., *V. deserticola*, Fig. 7A), or branched from the base (e.g., *V. transjordanicum*, Fig. 28A).

#### ﻿Leaves

**Figure 1.**
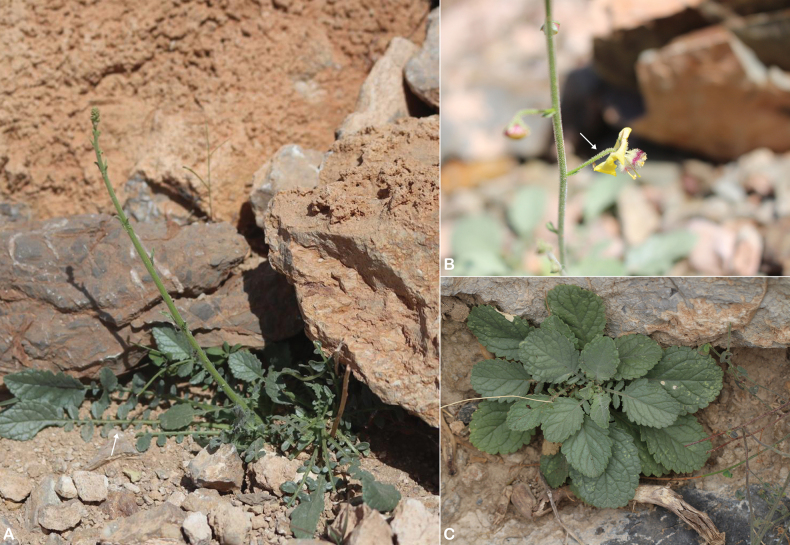
*Verbascum akdarense*. **A.** Habit; **B.** Flower and pedicel with glandular hairs (white arrow); **C.** Leaf. Photos: **A.** By Salim Al Rahbi; **B, C.** By Saif Al Hatmi.

In Arabian *Verbascum*, the leaves are usually arranged in a basal rosette, except in *V. saudiarabicum*, which has alternate basal leaves. Basal leaves are mostly oblong to oblong-lanceolate (e.g., *V. transjordanicum*, Fig. 28E), oblong to oblong-ovate (e.g., *V. sinaiticum*, Fig. 26B), oblong to obovate-oblong (e.g., *V. schimperianum*, Fig. 22A), oblong-ovate (e.g., *V. saudiarabicum*), oblanceolate (e.g., *V. virgatum*), lanceolate (e.g., *V. longibracteatum*), elliptic-lanceolate (e.g., *V. sarawaticum*, Fig. 20C), obovate (e.g., *V. akdarense*, Fig. 1C), obovate-elliptic to ovate (e.g., *V. shiqricum*, Fig. 24B), or obovate-oblong (e.g., *V. eremobium*). Cauline leaves can be decurrent (e.g., *V. medinecum*, Fig. 13F), sessile (e.g., *V. longibracteatum*), or sessile to petiolate (e.g., *V. sinaiticum*).

#### ﻿Indumentum

The indumentum is composed of three main types: glandular hairs, simple hairs, and branched hairs of various forms. A single *Verbascum* species may possess one (e.g., *V. schimperianum*), two (e.g., *V. deserticola*), or all three types (e.g., *V. transjordanicum*). These indumentum types also occur on the inner and outer surfaces of the corolla and on the outer surfaces of the calyx, pedicel, ovary, and capsule.

#### ﻿Inflorescence

Arabian *Verbascum* species exhibit racemose (e.g., *V. bottae*), dichasial (e.g., *V. eremobium*, Fig. 9A), and paniculate (e.g., *V. omanense*, Fig. 17A) inflorescence types. In addition, the number of flowers, including the presence of one or more accessory flowers, is useful for identification. These include single flowers lacking bracteoles (e.g., *V. decaisneanum*, Fig. 5B), single flowers with bracteoles (e.g., *V. eremobium*, Fig. 9C), clustered flowers lacking bracteoles (e.g., *V. shiqricum*), and clustered flowers with bracteoles (e.g., *V. longibracteatum*).

#### ﻿Calyx

Calyx lobes provide additional diagnostic characters for identifying Arabian *Verbascum* species. They may be oblong (e.g., *V. sarawaticum*, Fig. 20D), ovate-elliptic (e.g., *V. schimperianum*), linear (e.g., *V. shiqricum*), lanceolate (e.g., *V. sinaiticum*, Fig. 26D), oblanceolate (e.g., *V. virgatum*), or ovate-oblong (e.g., *V. akdarense*).

#### ﻿Corolla

The corolla is yellow, sometimes with markings on the throat (e.g., *V. shiqricum*), the upper side (e.g., *V. melhanense*, Fig. 15B), or both (e.g., *V. saudiarabicum*). The corolla typically has five petals but rarely has six or four in some individuals (e.g., *V. yemense* and *V. sinaiticum*). Another useful corolla character is the presence of pellucid glands, which can help delimit species, particularly those with overlapping geographical distributions (e.g., *V. sarawaticum*).

#### ﻿Stamens

*Verbascum* species possess four or five stamens (e.g., *V. melhanense* and *V. transjordanicum*, respectively, Figs 15B, 28B), which can be a valid diagnostic character when carefully assessed across populations. However, this character may be unstable in some species due to variation from four to seven stamens (e.g., *V. longibracteatum*, *V. yemense*, and *V. sinaiticum*, Figs 11C, 32D).

#### Filaments

In Arabian *Verbascum*, the distribution of filament indument shows consistent diagnostic patterns. In some species, filaments are densely hairy along their entire length up to the anthers (e.g., *V. omanense*, Fig. 17C). In others, the two anterior filaments become glabrous near the apex, whereas the posterior pair remains hairy to the top (e.g., *V. medinecum*, Fig. 13C). A third pattern occurs in species in which the two anterior filaments are completely glabrous, whereas the posterior filaments are fully pubescent (e.g., *V. melhanense*, Fig. 15B). Filament hair color is also variable and taxonomically informative, being white (e.g., *V. shiqricum*, Fig. 24C), yellow (e.g., *V. decaisneanum*, Fig. 5B), yellow-whitish (e.g., *V. medinecum*, Fig. 13B), yellowish-red (e.g., *V. saudiarabicum*), or red-purple (e.g., *V. eremobium*, Fig. 9B).

#### ﻿Anthers

Among Arabian species, three anther types are recognized: reniform anthers in the majority of *Verbascum* species; two anterior anthers inserted obliquely, occurring only in *V. saudiarabicum* and *V. virgatum* (Fig. 30C); and two anterior anthers inserted decurrently and longitudinally, found only in *V. bottae* and *V. melhanense* (Figs 3C, 15B).

#### ﻿Capsule

Capsule shapes in Arabian species are ellipsoid-ovoid (e.g., *V. yemense*), globose (e.g., *V. virgatum*), ovoid (e.g., *V. sarawaticum*), globose-subglobose (e.g., *V. eremobium*), globose-ovoid (e.g., *V. transjordanicum*), ellipsoid (e.g., *V. longibracteatum*, Fig. 11D), and pyriform-ovoid (e.g., *V. melhanense*, Fig. 15C).

#### ﻿Seeds

*Verbascum* species are divided into the sections Bothrosperma, characterized by transversely elongated seeds in most Arabian species, and Aulacospermae, characterized by longitudinally furrowed seeds found only in *V. saudiarabicum* and *V. bottae*, based on seed surface morphology, which is a distinct and stable character. In both sections, the seeds are oblong or ovoid in shape, brown or black in color, and approximately 1 mm in size.

### ﻿Taxonomic treatment

#### ﻿ Verbascum

Taxon classificationPlantaeLamialesScrophulariaceae

L., Sp. Pl. 1: 177 (1753)

036594E2-AEE0-595F-9672-7F1352104F7A

##### Type species.

*Verbascum thapsus* L.

#### ﻿ Celsia

Taxon classificationPlantaeLamialesScrophulariaceae

L., Sp. Pl. 2: 621 (1753)

B496754B-4D2D-5A24-8A96-477F063A90AC

##### Type species.

*Celsia orientalis* L.

#### ﻿ Staurophragma

Taxon classificationPlantaeLamialesScrophulariaceae

Fisch. & C.A.Mey., Index Seminum (LE, Petropolitanus) 9: 90 (1843)

2712E49C-D1BD-5039-BC93-365ADCBA7304

##### Type species.

*Staurophragma natolicum* Fisch. & C.A.Mey.

#### ﻿ Rhabdotosperma

Taxon classificationPlantaeLamialesScrophulariaceae

Hartl, Beitr. Biol. Pflanzen 53(1): 57 (1977)

95194534-3DFE-536E-AA68-FE99CBC5B87D

##### Type species.

*Rhabdotosperma brevipedicellatum* (Engl.) Hartl.

##### Description.

Annual, biennial, or perennial herbs to small shrubs, simple or branched from the base or above, sometimes with woody base, from 30 to 250 cm tall. *Indumentum* glabrescent, glandular hairs, simple hairs, and branched stellate hairs. *Stems* erect, simple or many stemmed, robust, terete, or terete to angular. *Basal and Cauline leaves* in rosettes or rarely alternate, mostly oblong to oblong-lanceolate or oblanceolate, sometimes obovate, elliptic, or ovate, with entire, dentate, repand-crenulate, undulate or lobed-crenate, serrate to sinuate, crenate to denticulate, sinuate, dentate, serrate, pinnatifid-lobed margins, sessile or petiole. *Inflorescence* racemose forming panicle or spike; one or more accessory flowers, panicle, dichasium, or racemose. *Upper and Lower bracts* linear, lanceolate, oblong to lanceolate or elliptic, triangular to ovate, ovate, or ovate to lanceolate, cordate to triangular, or oblanceolate. *Pedicel* hairy or glabrescent, from 2 to 25 mm long. ***Bracteoles*** absent or present ovate or to oblanceolate, lanceolate, ovate to triangular or lanceolate to triangular, linear, or lanceolate to cordate. ***Calyx*** lobes linear, oblong, oblong-lanceolate, ovate-oblong, lanceolate, ovate-elliptic, or oblanceolate, apex mucronate, acute, obtuse, or apiculate, hairy, or glabrescent. ***Corolla*** yellow with or without marks in the throat, sometimes on the upper side, with or without pellucid glands, tubeless or with a tube up to 2 mm, hairy or glabrous inside and outside. ***Stamens*** 4 or 5, sometimes unstable (4–5). ***Filaments*** with whitish, yellowish-purple, yellowish-red, yellow, purple-violet to violet-whitish, red-purple, yellowish-white, yellowish-violet to red, violet-whitish, or creamy hairs, with all hairs up to the anthers, two anterior glabrous, or two anterior glabrous near the apex. ***Anthers*** all reniform, two anterior inserted obliquely, or two anterior inserted decurrent longitudinally. ***Ovary*** pyriform-ovoid, ellipsoid, ellipsoid to ovoid, ovoid, globose, globose to ovoid, or globose-subglobose, hairy, or glabrous. ***Style*** from 4 to 22 mm long, filiform, green, or red. ***Stigma*** spatulate, capitate, dilated, or disciform. ***Capsule*** pyriform-ovoid, ellipsoid, ellipsoid to ovoid, ovoid, globose, globose to ovoid, or globose-subglobose, hairy, or glabrous. ***Seeds*** numerous, bothrospermous, or aulacospermous.

##### Distribution.

In Asia, Africa, and Europe, but the center of diversity is in Turkey and Iran. Elsewhere, some species, such as *Verbascum thapsus* L. and *V. blattaria* L., have become widely naturalized or invasive in many other temperate regions of the world, including North America, Australia, and New Zealand.

### ﻿Key to species of *Verbascum* in the Arabian Peninsula

**Table:** 

1	Seeds aulacospermous (longitudinally furrowed)	**2**
–	Seeds bothrospermous (transversely elongated)	**3**
2	Anthers two anterior inserted decurrent longitudinally	**2. *V. bottae***
–	Anthers two anterior inserted obliquely	**11. *V. saudiarabicum***
3	Flowers in clusters	**4**
–	Flowers solitary	**11**
4	Bracteoles present	**5**
–	Bracteoles absent	**9**
5	Anthers all reniform	**6**
–	Anthers two anterior inserted obliquely	**16. *V. virgatum***
6	Indumentum with sparse glandular-stellate hairs above and dense tomentose stellate hairs below	**9. *V. omanense***
–	Indumentum with dense tomentose stellate hairs above and below	**7**
7	Upper bracts ovate	**14. *V. sinaiticum***
–	Upper bracts linear	**8**
8	Cauline leaves with an obtuse base; leaves sessile	**6. *V. longibracteatum***
–	Cauline leaves with a cuneate base; leaves distinctly decurrent	**7. *V. medinecum***
9	Calyx lobes linear, apex acute, glabrescent, or sparse stellate	**10**
–	Calyx lobes oblong, apex mucronate, sparse stellate hairs	**10. *V. sarawaticum***
10	Basal leaves oblong-lanceolate, base obtuse or truncate, margins repand-crenulate	**17. *V. yemense***
–	Basal leaves obovate-elliptic to ovate, base obtuse to cuneate, margins crenate-sinuate	**13. *V. shiqricum***
11	Inflorescence racemose	**12**
–	Inflorescence dichasium	**5. *V. eremobium***
12	Anthers all reniform	**13**
–	Anthers two anterior inserted decurrent longitudinally	**8. *V. melhanense***
13	Stamens four	**14**
–	Stamens five	**16**
14	Capsule globose-ovoid	**15**
–	Capsule ovoid-ellipsoid	**4. *V. deserticola***
15	Indumentum dense glandular hairs above and simple hairs below	**1. *V. akdarense***
–	Indumentum dense glandular hairs above and stellate hairs below	**3. *V. decaisneanum***
16	Indumentum dense glandular hairs with sparse simple and stellate hairs above, and dense tomentose-stellate below	**15. *V. transjordanicum***
–	Indumentum dense rough tomentose-stellate above and below	**12. *V. schimperianum***

### ﻿Species descriptions

#### ﻿1. Verbascum akdarense

Taxon classificationPlantaeLamialesScrophulariaceae

(Murb.) Huber-Morath, Bauhinia 5(1): 10 (1973)

00255FF5-9526-50D1-B9CD-49316A60CFE6

Fig. 1

Celsia akdarensis Murb., in Lunds Univ. Arsskrift, n. f. xxii. No. 1, 123 (1925).

##### Type.

Oman, Muscat, Jabal Akhdar, *P.M.R. Aucher-Eloy 5044* (lectotype G [G00015116] designated here, isolectotype P [P03287290]).

##### Description.

Annual or biennial herb, pale green, simple or branched from the base, up to 45 cm tall. ***Indumentum*** dense glandular hairs above and pubescent hairs below. ***Stems*** erect, terete to angular. ***Basal leaves*** rosette, obovate, 3–10 × 2–5 cm, apex rounded, base cuneate, margins crenate-dentate, lamina darkish green with sparse glandular and dense pubescent hairs; petiole 2–5 cm, winged with a few small lateral lobes. ***Cauline leaves*** few or leafless, oblanceolate, 0.5–1.6 × 0.1–0.4 cm, apex obtuse, base obtuse; sessile. ***Inflorescence*** racemose; flowers single in the axil of bracts. ***Upper bracts*** lanceolate-linear, 1–3.5 mm, acute. ***Lower bracts*** lanceolate 2–5.5 mm, acute. ***Pedicel*** covered with dense glandular hairs up to 25 mm long. ***Bracteoles*** absent. ***Calyx*** 2–3 mm, lobes ovate-oblong, mucronate, dense glandular. ***Corolla*** 8–10 mm across, yellow with purple-red spots around the throat, without pellucid glands, tube up to 1 mm, sparse papillose-ciliated hairs inside, sparse glandular hairs outside. ***Stamens*** 4, 4–5 mm long. ***Filaments*** yellow with yellowish-purple hairs, two anterior glabrous near the apex, two posteriors with hairs up to the anthers. ***Anthers*** all reniform. ***Ovary*** globose, sparse glandular hairs. ***Style*** up to 6 mm long, filiform, green. ***Stigma*** capitate. ***Capsule*** 2–3 × 1–2 mm, globose-ovoid, sparse glandular hairy. ***Seeds*** bothrospermous.

##### Distribution in the Arabian Peninsula.

**Figure 2.**
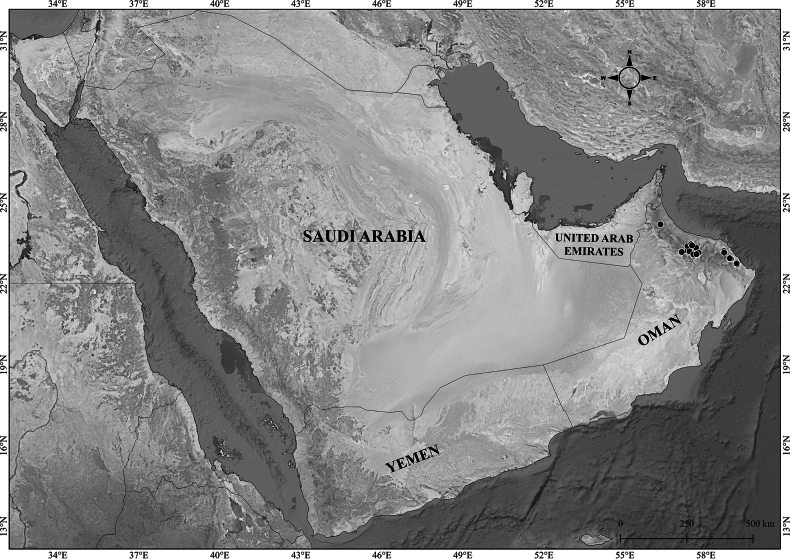
Distribution of *Verbascum akdarense* in the Arabian Peninsula.

It is an endemic species to Oman, which is known from Muscat (Jabal Aswad), Ash Sharqiyah North (Jabal Abyad, Jabal Bani Jabir, and near Tiwi), Ad Dakhiliyah (Jabal Al Akhdar, Wakan Village, Wadi Kamah, and Sakhakhin), and Al Batinah South (Wadi Bani Awf, Wadi Sahtan, An Nid, Wadi Haslah, and near Rustaq), northeast Oman (Fig. 2).

##### Ecology.

*Verbascum akdarense* grows on rocky slopes, rocks and fine soil, edge of wadis and gardens, and gravelly wadi beds at altitudes ranging from 200 to 2000 m. There are no records of associated plants.

##### Phenology.

Flowering and fruiting from March to May.

##### Etymology.

The name refers to Jabal Akhdar, known locally in Arabic as “al-Akhḍar.”

##### Specimens examined.

**Oman.** • **Muscat**: Jabal Aswad, above Siya, 21 March 1992, *I.S. Collenette 7994* (E [E00046311], ON); Northern, E of Hajar mountains, Siya at foot of Jabal Aswad, 30 September 1989, *A.G. Miller & J.A. Nyberg 9571* (E [E00066912]). • **Al Batinah South**: Vicinity of An Nid, 09 April 1975, *J.P. Mandaville 6403* (BM); vicinity of An Nid, 08 April 1975, *J.P. Mandaville 6303* & *6317* (BM); Wadi Sahtan, 05 April 1975, *J.P. Mandaville 6248* (BM); Wadi Haslah, SW of Awabi, 13 March 1978, *R.P. Whitcombe 150* (E [E00066911]); Al-Rustaq, 18 March 1975, *T.G. Rubens 94* (E [E00219516]); northern mountains, Wadi between Nakhl and Rustaq, 17 April 2001, *A. Patzelt 907* (K). • **Ad Dakhiliyah**: Wakan village, Northern Hajar mountains, 18 April 2007, *A. Patzelt 2441* (OBG); Wadi Kamah trail, 20 March 1972, *J.P. Mandaville 3663* (BM); upper end of Sakhakhin Gorge near Jabal Akhdar, 21 March 1976, *A. Radcliffe-Smith 3980* (BM, E [E00066951], K, ON); Jabal Akhdar, *P.M.R. Aucher-Eloy 5044* (G [G00015116], P [P03287290]). • **Ash Sharqiyah North**: Jabal Bani Jabir, Eastern Hajar mountains, 23 March 2009, *A. Patzelt 3771* (OBG); S of Tiwi, 25 March 1992, *I.S. Collenette 8026* (E [E00046276]).

##### Notes.

*Verbascum akdarense* is easily recognized by its indumentum of glandular and sparsely pubescent hairs, obovate basal leaves usually with a few small lateral lobes, and a globose-ovoid capsule. Phylogenetic analyses (Alzahrani et al. 2024) confirm it as a distinct species.

Murbeck (1925) cited multiple gatherings for this name and did not designate a holotype; all cited specimens therefore constitute syntypes. Among the original material, the specimen *Aucher-Eloy 5044* at G is the most complete and best preserved and matches the protologue. It is therefore selected here as the lectotype. The duplicate at P is treated as an isolectotype.

#### ﻿2. Verbascum bottae

Taxon classificationPlantaeLamialesScrophulariaceae

(Defl.) Huber-Morath, Bauhinia 5(1): 11 (1973)

C27B7C6D-091C-512B-BCF3-86AE7DC9DE03

Fig. 3

Rhabdotosperma bottae (Defl.) D.Hartl, Beitr. Biol. Pflanzen 53(1): 58 (1977).
Celsia bottae Defl., Voyage au Yemen, p. 178 (1889).

##### Type.

Yemen, Ad fauces jugi Hadhûr, prope Bauân, 30 June 1887, *A. Deflers 615* (lectotype MPU [MPU020118] designated here, isolectotype P [P03287260]).

##### Description.

Biennial herb, dark green to purple, simple or branched from above, woody at the base, up to 85 cm tall. ***Indumentum*** dense glandular hairs. ***Stems*** erect, robust, terete to angular. ***Basal leaves*** rosette, oblong to oblong-lanceolate, 4–15 × 1–5 cm, apex acute or obtuse, base subcordate-truncate, margins crenate-denticulate, lamina dark green with dense simple hairs on the veins below; petiole 2–6.5 cm, winged with 1–3 small lateral lobes. ***Cauline leaves*** lanceolate, 2–4.9 × 0.5–1 cm, apex acute, base subcordate-truncate; sessile. ***Inflorescence*** racemose; flowers single in the axil of bracts. ***Upper bracts*** lanceolate, 5–8 mm, acuminate. ***Lower bracts*** triangular-ovate, 14–45 mm, acuminate. ***Pedicel*** covered with dense glandular hairs up to 20 mm long. ***Bracteoles*** absent. ***Calyx*** 4–5.5 mm long, lobes oblong, mucronate, dense glandular hairs. ***Corolla*** 15–20 mm across, yellow with purple-red spots around the throat, without pellucid glands, tubeless, sparse papillose hairs inside, dense glandular hairs outside. ***Stamens*** 4, 7–8 mm long. ***Filaments*** red with yellowish-red hairs, two anterior glabrous near the apex, two shorter posteriors with hairs up to anthers. ***Anthers*** two anterior inserted decurrent longitudinally on filaments, two shorter posteriors with reniform anthers. ***Ovary*** pyriform-ovoid, glabrous. ***Style*** up to 15 mm long, filiform, green. ***Stigma*** dilated. ***Capsule*** 6–8 × 4–6 mm, pyriform-ovoid, glabrous. ***Seeds*** aulacospermous.

##### Distribution in the Arabian Peninsula.

It is an endemic species to Yemen, which is known from Ibb (Jabal Taqar, Sumara Pass, Jabal Sumara, and Jiblah town), Al Mahwit (Bait Albeshari), Sana’a (Khawlan, Jabal An Nabi Shu’ayb, and Jabal Shibam), Taizz (Turbah, Algaheli Park, and Jabal Sabr), Al-Bayda (Qarn Al-Wa’al), Hajjah (Kuslan town), and Raymah (Jabal Raymah), southwestern Yemen (Fig. 4).

##### Ecology.

*Verbascum bottae* grows on rocky slopes, limestone cliffs, terrace walls, granite crevices, and wadi banks at altitudes ranging from 1800 to 3100 m. There are no records of associated plants.

##### Phenology.

Flowering and fruiting from May to December.

##### Etymology.

The name commemorates Paul Émile Botta (1802–1870), a French naturalist and archaeologist.

##### Specimens examined.

**Yemen.** • Ad fauces jugi Hadhûr, prope Bauân, 30 June 1887, *A. Deflers 615* (MPU [MPU020118]). • **Ibb**: roadside S. of Ibb, 27 July 1973, *M. Brunt 2528* (K); on terrace walls, Jabal Taqar, 28 July 1977, *J.R.I Wood* 1707 (K); Sumara Pass, 26 March 1974, *J.J. Lavranos 11288* (E [E00066917], ON); Jiblah, environs of town, 16 October 1978, *A.G. Miller 536* (E [E00066921]); Sumara Pass, 09 October 1978, *A.G. Miller 462* (E [E00066918]); Jabal Sumara, 21 June 1979, *J.R.I Wood 2869* (BM, E [E00066922]). • **Al Mahwit**: Near Bait Albeshari, 14 December 1979, *J.R.I Wood 3108* (K). • **Sana’a**: By a water lake above Khawlan, 17 February 1978, *J.R.I Wood 2251* (K); Jabal An Nabi Shu’ayb, 30 September 1972, *J.R.I Wood 71* (BM); Jabal Shibam, 40 km N.E. of Sana’a, 17 October 1975, *F.N. Hepper 5768* (K); Jabal Shibam above Menacha, 05 October 1978, *A.G. Miller 377* (E [E00066914]); Shibam, 01 April 1981, *A.G. Miller* & *D.G. Long 3369* (E [E00066926]). • **Taizz**: Near Turbah, Algaheli, 10 November 1995, *M. Thulin, M. Ghebrehiwet* & *A.N. Gifri 9282* (K); Jabal Sabir, above and to the SE of Taizz, 03 August 1977, *A. Radcliffe-Smith* & *S.J. Henchie 4399* (K); west facing slopes of Jabal Sabir, 15 Km S of Taizz, 11 June 1982, *K.J. Gordon 1* (E [E00066923]); Turbah, 24 October 1974, *J.R.I Wood Y/74/155* (BM); Turbah, Jabal Sabir, 20 × 1974, *J.R.I Wood Y/74/166* (BM). • **Al-Bayda**: Qarn Al-Wa’al, 29 September 1976, *J.J. Lavranos* & *L.E. Newton 13045* (E [E00066916]). • **Hajjah**: Kuslan, 19 March 1979, *D. Wood Y1100* (E [E00066920]). • **Raymah**: Jabal Raymah, road from Al Jabin to Suq Ar Ribat, 22 March 1984, *A.G. Miller* & *R.A. King 5391* (E [E00687344]).

**Figure 3.**
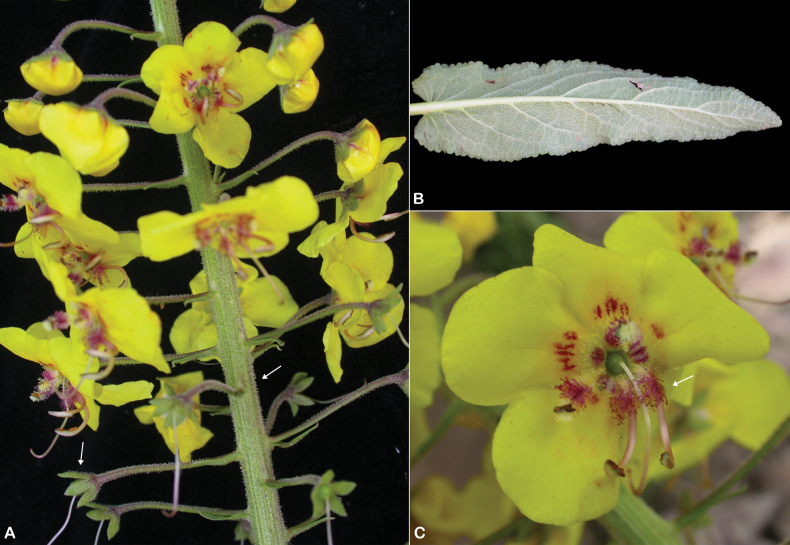
*Verbascum bottae*. **A.** Habit, calyx (white arrow), stems with glandular hairs (white arrow); **B.** Leaf; **C.** Filaments with two anterior anthers inserted decurrently and longitudinally and glabrous near the apex (white arrow). Photos by Abdul Wali Alkhulaidi.

##### Notes.

*Verbascum bottae* is easily confused with the morphologically similar *V. melhanense* but can be distinguished by its filament indumentum and seed morphology. *Verbascum bottae* has two anterior filaments that are glabrous near the apex and bears aulacospermous seeds, whereas *V. melhanense* has two anterior filaments that are completely glabrous and bothrospermous seeds. Recent phylogenetic research (Alzahrani et al. 2024) confirms that *V. bottae* is a distinct species. Deflers (1889) cited more than one specimen when describing this species without indicating a holotype. The specimen *Deflers 615* at MPU is complete, well preserved, and matches the protologue. It is therefore designated here as the lectotype. The duplicate at P is recognized as an isolectotype.

**Figure 4.**
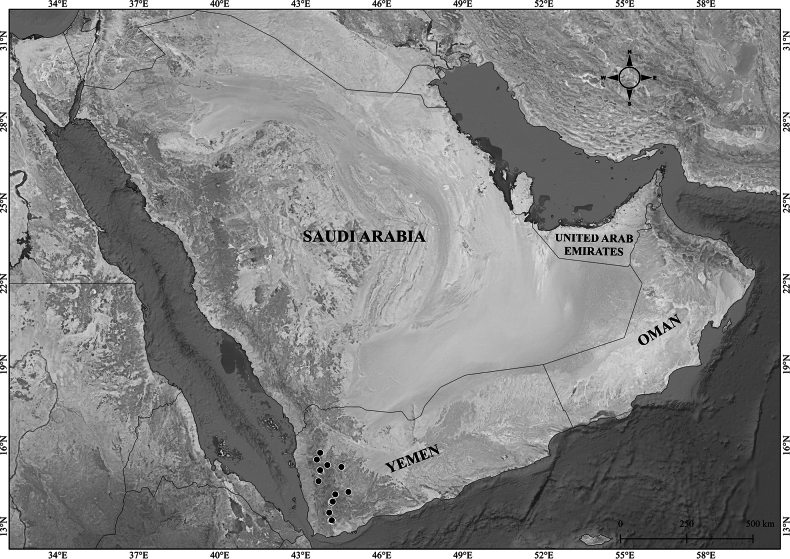
Distribution of *Verbascum bottae* in the Arabian Peninsula.

#### ﻿3. Verbascum decaisneanum

Taxon classificationPlantaeLamialesScrophulariaceae

O.Kuntze, Revis. Gen. Pl. 2: 468 (1891)

63C18022-68F1-5C3B-A815-B99B8D09B641

Fig. 5

Celsia parviflora Decne., Ann. Sci. Nat., Bot. sér. 2, 2: 254 (1834).

##### Type.

Egypt, ad latus septentrionale montis St. Catharinae, 20 June 1835, *W. Schimper 282* (lectotype HBG [HBG511640] designated here, isolectotype HBG [HBG511642]).

##### Description.

Perennial herb, brownish green, very branched from the base, many-stemmed, woody at the base, up to 60 cm tall. ***Indumentum*** dense glandular hairs above and forked hairs below. ***Stems*** erect, terete. ***Basal leaves*** rosette, oblong-lanceolate, 3–5 × 1.5–2 cm, apex acute, base attenuate, margins entire or dentate, lamina yellowish green with sparse glandular and dense forked hairs; petiole 0.5–1.5 cm. ***Cauline leaves*** few or leafless, linear-lanceolate, 0.5–0.7 × 0.1–0.2 cm, apex acute, base attenuate; sessile. ***Inflorescence*** racemose; flowers single in the axil of bracts. ***Upper bracts*** linear-lanceolate, 0.5–1.8 mm, acute. ***Lower bracts*** linear-lanceolate, 0.5–2 mm, acute. ***Pedicel*** covered with dense glandular and sparse forked hairs up to 10 mm long. ***Bracteoles*** absent. ***Calyx*** 1–3 mm, lobes lanceolate, acute, dense glandular and sparse forked. ***Corolla*** 8–10 mm across, yellow with a red spot around the throat, without pellucid glands, tube up to 2 mm, sparse papillose-ciliated hairs inside, sparse glandular and forked hairs outside. ***Stamens*** 4, 4–6 mm long. ***Filaments*** yellow with yellow hairs, two anterior glabrous near the apex, two posteriors with hairs up to anthers. ***Anthers*** all reniform. ***Ovary*** globose-ovoid, sparse glandular and forked hairs. ***Style*** up to 7 mm long, filiform, green. ***Stigma*** capitate. ***Capsule*** 2–3 × 1.5–2 mm, globose-ovoid, sparse glandular and forked hairy. ***Seeds*** bothrospermous.

**Figure 5.**
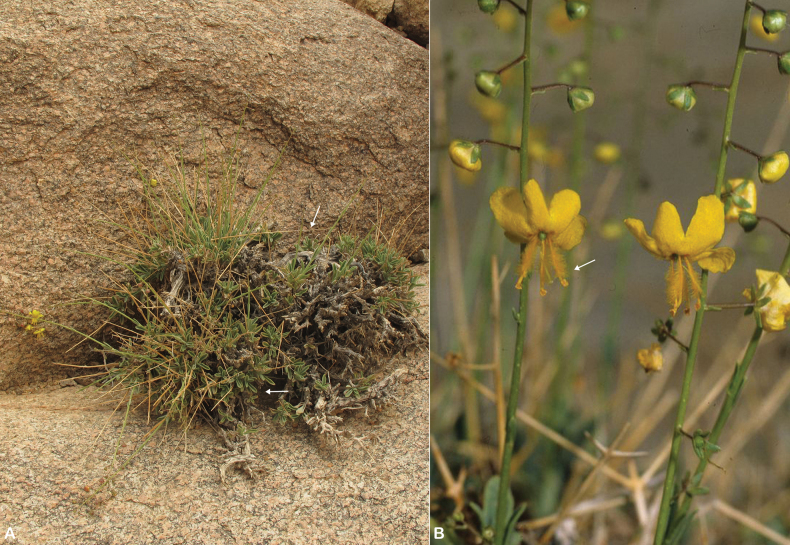
*Verbascum decaisneanum*. **A.** Habit showing woody base (white arrows); **B.** Flowers with four stamens (white arrow). Photos: **A.** By Tony Miller; **B.** By Sheila Collenette.

##### Distribution.

Lebanon, Jordan, Syria, Palestine, Egypt (Sinai), and Saudi Arabia.

##### Distribution in the Arabian Peninsula.

**Figure 6.**
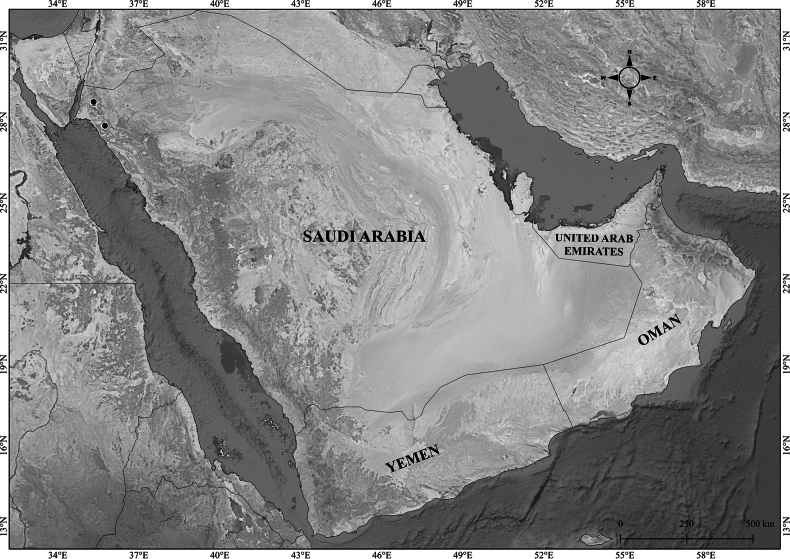
Distribution of *Verbascum decaisneanum* in the Arabian Peninsula.

It is a native species to Saudi Arabia, which is known from two locations in Tabuk province (Jabal Al-Lawz and Jabal Dabbagh), northwest Saudi Arabia (Fig. 6).

##### Ecology.

*Verbascum decaisneanum* grows in granite crevices of cliffs and rocky slopes at altitudes ranging from 1500 to 1900 m. Associated plants include *Dianthus sinaicus* Boiss., *Hypericum sinaicum* Hochst. ex Boiss., *Kickxia collenetteana* D.A.Sutton, *Lactuca orientalis* (Boiss.) Boiss., *Phlomis brachyodon* (Boiss.) Zohary ex Rech.f., *Pistacia khinjuk* Stocks, *Pterocephalus sanctus* Decne., and *Verbascum sinaiticum* Benth.

##### Phenology.

Flowering and fruiting from April to June.

##### Etymology.

The name commemorates Joseph Decaisne (1807–1882), a French botanist and plant illustrator.

##### Specimens examined.

**Egypt.** • **Septentrionale**: montis St. Catharinae, 20 June 1835, *W. Schimper 282* (HBG [HBG511640] & [HBG511642]).

**Saudi Arabia.** • **Tabuk**: North Hijaz, Jabal Dabbagh, 04 May 1978, *I.S. Collenette* 717 (K); Jabal Al-Lawz, south of Aqaba, 05 May 1992, *I.S. Collenette 8204* (K, E [E01000561]); Jabal Dabbagh, 100 km SW of Tabuk, 11 April 1985, *I.S. Collenette 5260* (E [E00066909]).

##### Notes.

*Verbascum decaisneanum* is distinct in having dense glandular hairs above and forked hairs below, oblong-lanceolate basal leaves, four stamens, and yellow filaments with yellow hairs. Phylogenetic analyses (Alzahrani et al. 2024) support its recognition as a distinct species.

The protologue of this species cites *Schimper 282* but does not indicate a single type specimen. Duplicate material exists in multiple herbaria. The HBG sheet selected as the lectotype is the most complete and clearly displays the diagnostic characters noted in the protologue.

#### ﻿4. Verbascum deserticola

Taxon classificationPlantaeLamialesScrophulariaceae

(Murb.) Huber-Morath, Bauhinia 5(1): 12 (1973)

B2F1D09B-3CEA-52F9-98A3-84D03ABDDB8F

Celsia deserticola Murb., in Lunds Univ. Arsskrift, n. f. xxii. No. 1, 92 (1925).

##### Type.

Saudi Arabia, Bir Neghile bei Moileh, July 1825, *Ehrenberg*, s.n. (B†). Saudi Arabia, 75 km E of Duba to Shiqri road, 01 April 1989, *I.S. Collenette 7060* (neotype E [E00066907] designated here, isoneotype K).

### ﻿Key to the varieties

**Table:** 

1	Basal leaves ovate-obovate to lanceolate, apex acute or mucronate, base rounded or obtuse, margins serrate to sinuate, lamina yellowish or grey green with rough tomentose with stellate hairs; petiole 1–6.5 cm	**4a. V. deserticola var. deserticola**
–	Basal leaves oblong-lanceolate, apex acute, base obtuse, margins deep crenate to sinuate, lamina yellowish green with dense stellate hairs; petiole 0.5–1.5 cm	**4b. V. deserticola var. sheilae**

#### ﻿4a. Verbascum deserticola var. deserticola

Taxon classificationPlantaeLamialesScrophulariaceae

72C25418-E70D-5ED1-B873-147EF680E355

Fig. 7

##### Description.

Biennial herb, yellowish or greyish green, branched from above, woody at the base, up to 65 cm tall. ***Indumentum*** dense glandular hairs above, and dense tomentose with stellate hairs below. ***Stems*** erect, terete. ***Basal leaves*** rosette, ovate-obovate to lanceolate, 3–15 × 1.5–6 cm, apex acute or mucronate, base rounded or obtuse, margins serrate to sinuate, lamina yellowish or grey green with rough tomentose with stellate hairs; petiole 1–6.5 cm. ***Cauline leaves*** oblong-lanceolate or lanceolate, 2–4.9 × 0.5–1 cm, apex acute, base semi-amplexicaul; sessile or petiole up to 1 cm. ***Inflorescence*** racemose, a single flower in the axil of the bract. ***Upper bracts*** lanceolate, 1.6–2.8 mm, acute. ***Lower bracts*** ovate or ovate-lanceolate, 6–17 mm, acute or mucronate. ***Pedicel*** covered with glandular hairs, up to 8 mm long. ***Bracteoles*** absent. ***Calyx*** 1.5–3 mm, lobes oblong to oblong-lanceolate, obtuse, mucronate to apiculate, glandular hairs. ***Corolla*** 8–10 mm across, yellow with a purple-red spot around the throat, without pellucid glands, tube up to 1 mm, sparse papillose-ciliated hairs inside, glandular hairs outside. ***Stamens*** 4, 5–8 mm long. ***Filaments*** red with purple-violet to violet-whitish hairs, two anterior glabrous near the apex, two posteriors with hairs up to anthers, all reniform anthers. ***Ovary*** ovoid, sparse glandular hairs. ***Style*** up to 8 mm long, filiform, green, or violet. ***Stigma*** capitate. ***Capsule*** 2.8–3 × 2–2.7 mm, ovoid-ellipsoid, glabrous. ***Seeds*** bothrospermous.

##### Distribution in the Arabian Peninsula.

It is an endemic species to Saudi Arabia, which is known from several locations in Tabuk province (Tabuk road between Duba and Shigry and near Jabal Shar), northwest Saudi Arabia, as well as from several locations in Medina province (Jabal Radwa, Road to Jabal Al-Figrah, Wadi Buwat, and between Al Wajh and Al-Ula), western Saudi Arabia (Fig. 8).

##### Ecology.

*Verbascum deserticola* grows on rocky black hillsides, rocky slopes, roadsides, and among fallen rocks in wadis at altitudes ranging from 400 to 1300 m. Associated plants include *Cleome droserifolia* (Forssk.) Delile, *Kickxia aegyptiaca* (L.) Nábělek, *Nerium oleander* L., *Plocama calycoptera* (Decne.) M.Backlund & Thulin, *Reichardia tingitana* (L.) Roth, Vachellia tortilis subsp. raddiana (Savi) Kyal. & Boatwr., and Vachellia tortilis subsp. tortilis.

##### Phenology.

Flowering and fruiting from March to November.

##### Etymology.

The name refers to its occurrence in desert habitats.

##### Specimens examined.

**Saudi Arabia.** • **Tabuk**: 75 km E of Duba to Shiqri road, 03 August 1989, *I.S. Collenette 7237* (E [E00066908], K); 75 km E of Duba to Shiqri road, 01 April 1989, *I.S. Collenette 7060* (E [E00066907], K); Tabuk road between Duba and Shigry, near Ras Al-Khuraytah primary school, 02 March 2021, *A. Alzahrani 147* (MUZ). • **Medina**: Jabal Radwa, 01 January 1983, *D. Lickfold 8752* (RIY); Jabal Radwa, 100 km N of Yanbo, Wadi on N side, 15 August 1982, *I.S. Collenette 3802* (E [E00066910], K); Jabal Radwa, N of Yanbo, 10 October 1981, *I.S. Collenette 2908* (E [E00687348], K); Somewhere N of Al Wajh to Al-Ula, dirt track, 18 March 1986, *I.S. Collenette 5767* (E [E00066965], K, RIY); Wadi Buwat, between Medina and Yanbo Al-Nakal, 06 May 1992, *I.S. Collenette 8215* (K); Road to Jabal Al-Figrah, 07 March 2021, *A. Alzahrani 152* (MUZ).

**Figure 7.**
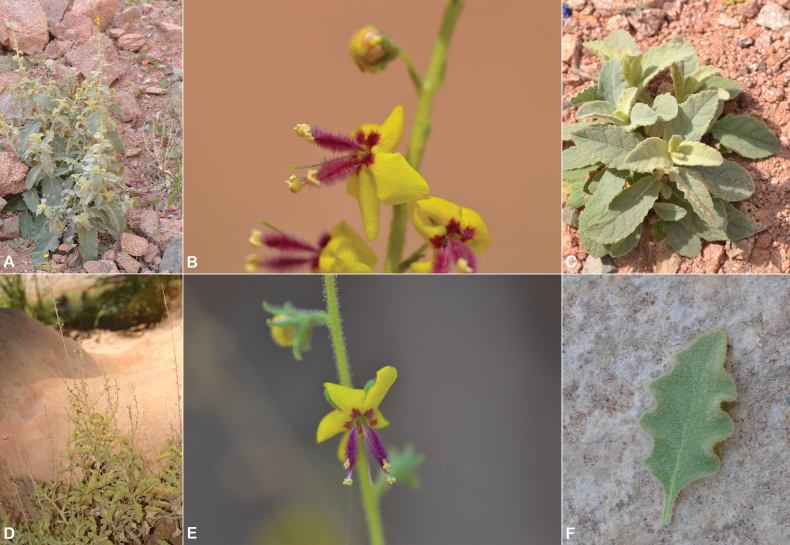
Verbascum deserticola var. deserticola. **A.** Habit; **B.** Flowers with four stamens; **C.** Leaf. V. deserticola var. sheilae. **D.** Habit; **E.** Flowers with four stamens; **F.** Leaf. Photos by Ali Alzahrani.

##### Notes.

*Verbascum deserticola* is a highly variable species that is widespread in western and northwestern Saudi Arabia. Al-Hemaid (2001) separated *V. sheilae* as a distinct species based on this variation. Phylogenetic evidence (Alzahrani et al. 2024), together with a comparative study of the type specimens, shows that both names refer to the same taxon. Diagnostic characters shared by both include a woody base, dense glandular hairs above, dense tomentose stellate hairs below, four stamens, a racemose inflorescence with solitary flowers, purple-violet to whitish filament hairs, and a glabrous ovoid-ellipsoid capsule. *Verbascum sheilae* is therefore treated here as a variety of *V. deserticola*.

The original type was destroyed in Berlin, and no original material survives despite an exhaustive search. To ensure nomenclatural stability, *Collenette 7060* is designated here as the neotype because it matches the diagnostic characters in Murbeck’s protologue and was collected near the original type locality.

**Figure 8.**
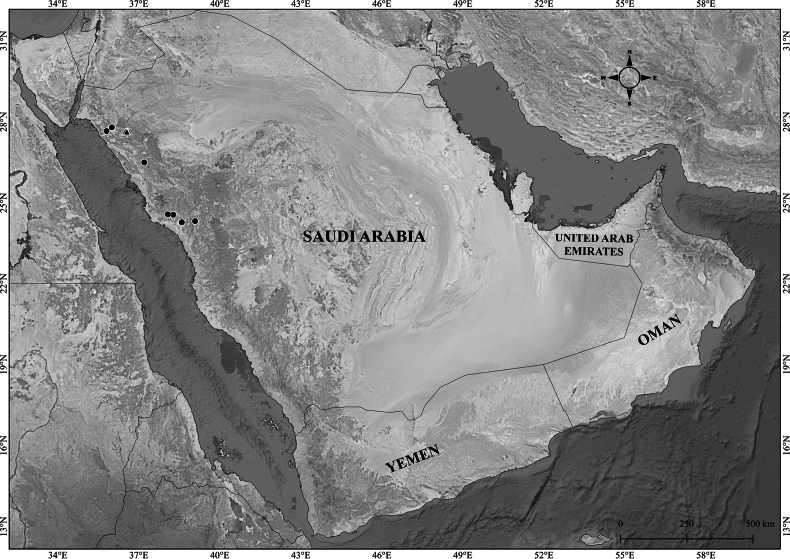
Distribution of Verbascum deserticola var. deserticola (black circles) and V. deserticola var. sheilae (black triangles) in the Arabian Peninsula.

#### ﻿4b. Verbascum deserticola var. sheilae

Taxon classificationPlantaeLamialesScrophulariaceae

(Hemaid) A.Alzahrani stat. nov.

C4AD6B71-1DF6-57D2-80DE-816C97F735F9

urn:lsid:ipni.org:names:77374373-1

Fig. 7

Verbascum sheilae Hemaid, Pakistan J. Bot. 33(4): 324 (2001).

##### Type.

Saudi Arabia, Tabuk, Headwater of Wadi Qaraqir, E of Duba, 17 March 1994, *I.S. Collenette 9072* (holotype K, isotype E [E00092232] & [E00092213]).

##### Description.

Biennial herb, yellowish or greyish green, branched from above, woody at the base, up to 65 cm tall. ***Indumentum*** dense glandular hairs above, and dense tomentose with stellate hairs below. ***Basal leaves*** rosette, oblong-lanceolate, 3–10 × 1.5–6 cm, apex acute, base obtuse, margins deep crenate to sinuate, lamina yellowish green with dense stellate hairs; petiole 0.5–1.5 cm. ***Cauline leaves*** oblong-lanceolate or lanceolate, 2–4.9 × 0.5–1 cm, apex acute, base semi-amplexicaul; sessile or petiole up to 1 cm. ***Inflorescence*** as for var. deserticola.

##### Distribution in the Arabian Peninsula.

It is known from one location in Tabuk province (Wadi Al-Disah), northwest Saudi Arabia (Fig. 8).

##### Etymology.

The name commemorates Sheila Collenette (1927–2017), a British botanist and plant collector who made significant contributions to the study of the flora of Saudi Arabia.

##### Specimens examined.

**Saudi Arabia.** • **Tabuk**: Wadi Al-Lawz off wadi Qaraqir, E of Duba, 25 August 1994, *I.S. Collenette 9154* (E [E00095075], K); Wadi Ghamrah off wadi Qaraqir, E of Duba, 25 August 1994, *I.S. Collenette 9153* (E [E00095076], K); Headwater of Wadi Qaraqir, E of Duba, 17 March 1994, *I.S. Collenette 9072* (E [E00092232] & [E00092213], K, RIY); Wadi Disah, wadi Qaraqir, 08 March 2013, *J. Thomas 23970* (KSU); Wadi Disah, SW of Tabuk, 01 April 2014, *J. Thomas 23742* (KSU); Wadi Al-Disah, 21 August 2020, *A. Alzahrani 86* (MUZ); Wadi Al-Disah, 21 August 2020, *A. Alzahrani 85* (MUZ).

##### Notes.

This variety differs from var. deserticola in having oblong-lanceolate leaves, deeply crenate to sinuate margins, a yellowish-green lamina with dense stellate hairs, and a 0.5–1.5 cm petiole. It is restricted to Wadi Al-Disah in the Tabuk region of northwestern Saudi Arabia.

#### ﻿5. Verbascum eremobium

Taxon classificationPlantaeLamialesScrophulariaceae

Murb. in Lunds Univ. Arsskrift, N. F. xxix. No. 2 p. 458 (1933)

AD13AC8A-DD96-52EA-9322-3A524A5A91A6

Fig. 9

Verbascum tabukum Hemaid, Pakistan J. Bot. 33(4): 327 (2001), syn. nov. – Type. Saudi Arabia, Tabuk, Duba road, 28 April 1994, *I.S. Collenette 9115* (holotype K).

##### Type.

Syria, Palmyre, 30 April 1928, *R. Gombault 462* (lectotype P [P03787920] designated here).

##### Description.

Perennial herb, yellowish or greyish green, very branched from the base, woody at the base, up to 75 cm tall. ***Indumentum*** dense rough tomentose with stellate hairs. ***Stems*** erect, terete. ***Basal leaves*** rosette, obovate-oblong, 5–10 × 2–5 cm, apex obtuse, base obtuse to cuneate, margins undulate or lobed-crenate, lamina yellowish or grey green with dense yellowish or greyish green tomentose with stellate hairs; petiole 2–5 cm. ***Cauline leaves*** oblong-obovate to ovate, 3–6 × 2–3 cm, apex acute, base semi-amplexicaul; sessile. ***Inflorescence*** dichasium; three-flowered, or one flowered, peduncle in the axil of bracts. ***Upper bracts*** lanceolate, 2–3 mm, acute. ***Lower bracts*** cordate-triangular or triangular, 20–50 mm, acute. ***Pedicel*** covered with dense tomentose with stellate hairs up to 5 mm long. ***Bracteoles*** present, lanceolate-cordate, acute. ***Calyx*** 5–8 mm, lobes lanceolate, acute, dense tomentose with stellate. ***Corolla*** 15–20 mm across, yellow with red blotches around the throat, with pellucid glands, tube up to 1 mm, glabrous inside, dense tomentose with stellate hairs outside. ***Stamens*** five, 4–6 mm long. ***Filaments*** red with red-purple hairs, two anterior glabrous near the apex, three posteriors with hairs up to anthers. ***Anthers*** all reniform. ***Ovary*** globose, dense tomentose with stellate hairs. ***Style*** up to 6 mm long, filiform, green. ***Stigma*** capitate. ***Capsule*** 3–4 × 3–5 mm, globose-subglobose, dense tomentose with stellate hairs. ***Seeds*** bothrospermous.

##### Distribution.

Lebanon, Jordan, Syria, Jordan, Palestine, Egypt (Sinai), and Saudi Arabia.

##### Distribution in the Arabian Peninsula.

It is a native species to Saudi Arabia, which is known from several locations in Tabuk province (Jabal Thaghb, near Jabal Al-Lawz road, Jalah, near Wadi Sadrr, near Duba, and Wadi Aba Al-Hinshan), northwest Saudi Arabia (Fig. 10).

##### Ecology.

*Verbascum eremobium* grows on rocky black hillsides, barren rocky wadis, and roadsides at altitudes ranging from 915 to 1420 m. Associated plants include *Argyrolobium crotalarioides* Jaub. & Spach, *Diplotaxis harra* (Forssk.) Boiss., *Linaria haelava* (Forssk.) Delile, *Morettia canescens* Boiss., *Onopordum ambiguum* Fresen., *Vachellia gerrardii* (Benth.) P.J.H.Hurter, and *Zygophyllum molle* (Delile) Christenh. & Byng.

**Figure 9.**
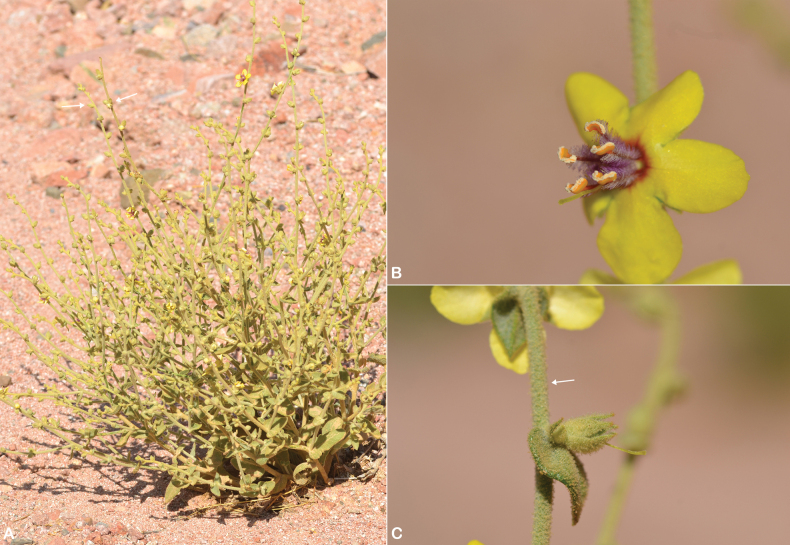
*Verbascum eremobium*. **A.** Habit and dichasial inflorescence (one- and three-flowered, white arrows); **B.** Flowers with five stamens; **C.** Calyx and stems with dense, rough, tomentose stellate hairs. Photos by Ali Alzahrani.

##### Vernacular name.

*Desert mullein* (English).

##### Phenology.

Flowering and fruiting from April to November.

##### Etymology.

The name refers to its adaptation to desert habitats.

##### Specimens examined.

**Saudi Arabia.** • **Tabuk**: Duba road, 28 April 1994, *I.S. Collenette 9115* (E [E00092230 & E00092231], K); near Shiqri, Tabuk road, 20 April 1983, *I.S. Collenette 4347* (E [E00066929]); near Jabal Al-Lawz, 28 March 1989, *I.S. Collenette 7048* (E [E00066928], K); 8 km south of Jabal Al-Lawz, 2 August 1989, *I.S. Collenette 7227* (E [E00066930], K).

**Syria.** • **Palmyre**: 30 April 1928, *R. Gombault 462* (P [P03787920]).

• **Transjordanien**: 9 April 1932, *R. Gombault 19* (P [P03285763]).

##### Notes.

*Verbascum tabukum* was described by Al-Hemaid (2001) based on its four stamens, sessile flowers, and ovate leaves. However, phylogenetic analyses (Alzahrani et al. 2024) place it within *V. eremobium*. Examination of the type specimens of *V. tabukum* and *V. eremobium*, together with comparison of key morphological traits, shows that both taxa share a woody base, dense rough stellate tomentum, obovate-oblong basal leaves, five stamens, dichasial inflorescences, one or three flowers per node, red-purple filament hairs, and a globose to subglobose capsule. Their similar geographic distributions in Saudi Arabia and neighboring regions further support their conspecificity. *V. tabukum* is therefore treated as a synonym of *V. eremobium*.

**Figure 10.**
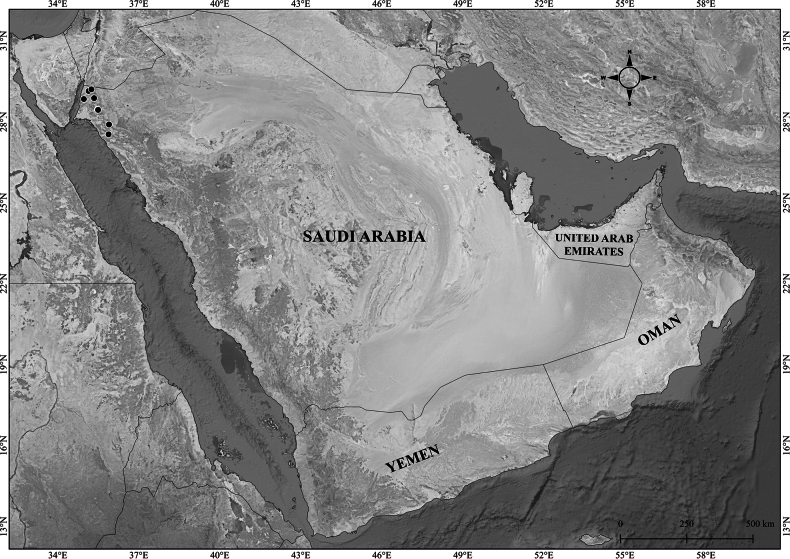
Distribution of *Verbascum eremobium* in the Arabian Peninsula.

The original material cited by Murbeck includes several specimens. To ensure nomenclatural stability, the specimen *Gombault 462* housed at P is designated here as the lectotype because it best corresponds to the protologue and represents the most complete and well-preserved original material available.

#### ﻿6. Verbascum longibracteatum

Taxon classificationPlantaeLamialesScrophulariaceae

Defl., Bull. Soc. Bot. France 43: 218 (1896)

FA3B53DA-9224-56A2-ABFF-F6948D352DFC

Fig. 11

Verbascum luntii Baker, Bull. Misc. Inform. Kew (93): 337 (1894), syn. nov. – Type: Yemen, Hillsides at Alrail, 28 December 1893, *W. Lunt 119* (lectotype K [K000975903] designated here).

##### Type.

Yemen, Bilad Fodhli, ad fauces australes montis el-’Areys, circa Serrya, 24 April 1893, *A. Deflers 868* (lectotype G [G00343570] designated here, isolectotypes MPU [MPU020131] & [MPU020130]).

##### Description.

Perennial herb, yellowish green, simple, or usually branched from above, up to 2 m tall. ***Indumentum*** dense tomentose with stellate hairs. ***Stems*** erect, robust, terete. ***Basal leaves*** rosette, lanceolate, 10–20 × 3–8 cm, apex acute, base obtuse, margins crenate, lamina yellowish green with densely tomentose with stellate hairs; petiole 2–4 cm. ***Cauline leaves*** lanceolate, 4–8 × 1–3 cm, apex acute, base obtuse; sessile. ***Inflorescence*** panicle; with clusters of 2–8 flowers in the axil of bracts. ***Upper bracts*** linear, 5–15 mm, acute. ***Lower bracts*** linear, 30–50 mm, acute. ***Pedicel*** covered with densely tomentose with stellate hairs up to 6 mm long. ***Bracteoles*** present, linear, acute. ***Calyx*** 4–7 mm, lobes linear, acute, densely tomentose with stellate hairs. ***Corolla*** 15–20 mm across, yellow, without pellucid glands, tube up to 2 mm, densely tomentose with stellate hairs outside. ***Stamens*** 4 – 5 or 7 (unstable), 5–6 mm long. ***Filaments*** orange to yellow with yellowish-white hairs, two anterior glabrous near the apex, three posteriors with hairs up to anthers. ***Anthers*** all reniform. ***Ovary*** ellipsoid, dense tomentose with stellate hairs. ***Style*** up to 8 mm long, filiform, green. ***Stigma*** spatulate. ***Capsule*** 4–8 × 3–4 mm, ellipsoid, dense tomentose with stellate hairs. ***Seeds*** bothrospermous.

**Figure 11.**
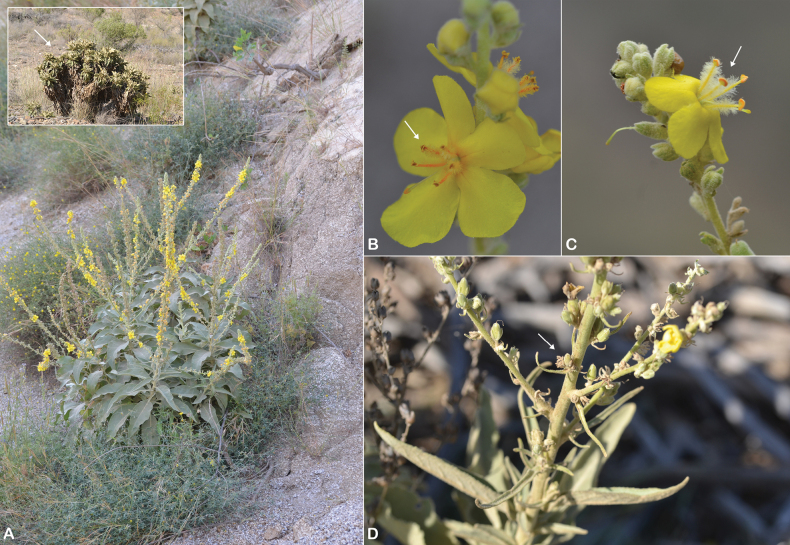
*Verbascum longibracteatum*. **A.** Habit showing massive growth (white arrow); **B, C.** Flowers with five and four stamens, respectively; **D.** Bracts and capsules (white arrow). Photos by Ali Alzahrani.

##### Distribution in the Arabian Peninsula.

**Figure 12.**
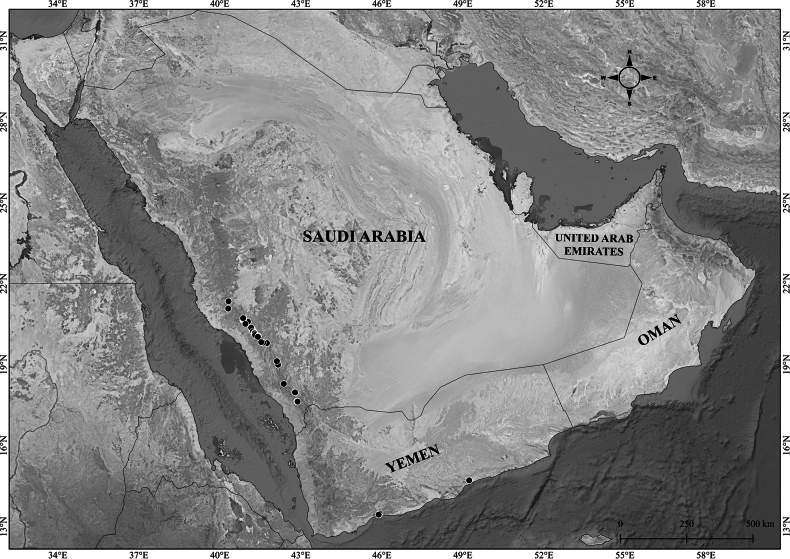
Distribution of *Verbascum longibracteatum* in the Arabian Peninsula.

It is an endemic species to the Arabian Peninsula, which is known from Yemen in Abyan (Jabal Areys) and Hadhramaut (Alrail), and from Saudi Arabia in Taif (Red Mountain and near Al-Hada), Al-Baha (Al-Abna Road, Heznah Road, and near Wadi Shora), Abha (Jabal Al-Soudah, Wadi Namra, and near Tanomah), and Jizan (Jabal Al-Qahar), in southwestern Arabian Peninsula (Fig. 12).

##### Ecology.

*Verbascum longibracteatum* grows on roadsides, rocky slopes, and edges of wadis at altitudes ranging from 300 to 2750 m. Associated plants include *Anagyris foetida* L., *Astragalus atropilosulus* (Hochst.) Bunge, *Coleus arabicus* Benth., *Juniperus procera* Hochst. ex Endl., *Pentas lanceolata* (Forssk.) Deflers, *Rumex nervosus* Vahl, *Rumex vesicarius* L., *Vachellia johnwoodii* (Boulos) Ragup., Seigler, Ebinger & Maslin, and *Vachellia origena* (Hunde) Kyal. & Boatwr.

##### Vernacular name.

*Zohara* (Arabic).

##### Phenology.

Flowering and fruiting from March to May.

##### Etymology.

The name refers to the species’ long bracts, a distinctive characteristic.

##### Specimens examined.

**Saudi Arabia.** • **Abha**: Jabal Soudah, northwest of Abha, 10 March 1980, *I.S. Collenette 2049* (K); near Al Hasane, 98 km from the Abha to Najran road, 22 November 1985, *I.S. Collenette 5525* (E [E00066946]); near Tanomah, 02 May 1985, *I.S. Collenette 7170* (K); between Abha and Jabal Soudah, 17 October 1981, *I.S. Collenette 2964* (E [E00066947]); Wadi Namra, 23 April 1982, *A.C. Podzorski 1068* (E [E00687345]). • **Al-Baha**: Al-Abna Road, 7 km S of Baljurashi, 24 February 1994, *I.S. Collenette 9015* (K); Al-Abna Road, 5 km S of Baljurashi, 17 April 1983, *I.S. Collenette 4330* (K); between Baljurashi and Maquas, 08 August 1982, *P. König & H. Kürschner 82/2125* (E [E00687346]); Heznah Road between Al-Makhwah and Baljurashi, 15 March 2021, *A. Alzahrani 170* (MUZ); Al-Abna road between Al-Awamer and Baljurashi, 15 March 2021, *A. Alzahrani 171* (MUZ); Al-Baha – Abha road, near Wadi Shora, Baljurashi, 13 March 2021, *A. Alzahrani 167* (MUZ). • **Jizan**: Jabal Al-Qahar, 07 May 1990, *I.S. Collenette 7544* (K); Jabal Al-Qahar, 5500 ft, 29 April 1989, *I.S. Collenette 7157* (K); Jabal Al-Qahar, 12 February 2021, *A. Alzahrani 144* (MUZ). • **Taif**: 5 km southwest of Al-Hada, 26 March 1979 *I.S. Collenette 1090* (K); Al-Hada, 20 March 1980 *I.S. Collenette 2169* (K); third way of Jeddah Taif Road, 19 February 1980 *I.S. Collenette 1891* (K); Red Mountain, near Al-Hada, 10 February 1982, *I.S. Collenette 3222* (E [E00066945], K).

**Yemen.** • **Abyan**: Jabal Areys, 11 February 1989, *A.G. Miller, L. Guarino, N. Obadi, M. Hassan & N. Mohammed 8103* (E [E00066963]); Bilad Fodhli, ad fauces australes montis el-’Areys, circa Serrya, 24 April 1893, *A. Deflers 868* (isosyn. MPU [MPU020130]); Bilad Fodhli, in Wadi el-’Areys (6 km ad orient. urb. Schughra), 23 March 1890, *A. Deflers 441* (isosyn. MPU [MPU020132]). • **Hadhramaut**: Hillsides at Alrail, 28 December 1893, *W. Lunt 119* (K [K000975903]).

##### Notes.

Deflers (1896) cited several gatherings in the protologue; all constitute original material. Among these, the specimen *Deflers 868* preserved at G is designated here as the lectotype because it is the most complete and best matches the diagnostic features given in the protologue. The duplicates at MPU are treated as isolectotypes.

#### ﻿7. Verbascum medinecum

Taxon classificationPlantaeLamialesScrophulariaceae

Hemaid, Pakistan J. Bot. 33(4): 321 (2001)

F682FC16-6A2A-5C12-80C0-EF9FAF203172

Fig. 13

Verbascum hema-figranum Hemaid, Pakistan J. Bot. 33(4): 321 (2001), syn. nov. – Type: Saudi Arabia, Hema Al-Figrah, Medina, 02 March 1989, *I.S. Collenette 6977* (holotype E [E00066970]).

##### Type.

Saudi Arabia, Hema Al-Figrah, 60 km W of Medina, 20 April 1989, *I.S. Collenette 7116* (holotype K, isotype E [E00066952]).

##### Description.

Perennial herb, yellowish or greyish green, simple or few short branched above, up to 1.5 m tall. ***Indumentum*** dense tomentose with stellate hairs. ***Stems*** erect, robust, terete. ***Basal leaves*** rosette, oblong-lanceolate, 10–25 × 3–6 cm, apex acute, base cuneate, margins crenate-sinuate, lamina yellowish or greyish green with dense tomentose with stellate hairs; petiole 3–7 cm. ***Cauline leaves*** lanceolate, 3–7 × 1–3 cm, apex acute, base cuneate; decurrent. ***Inflorescence*** panicle; one or clusters of 2–8 flowers in the axil of bracts. ***Upper bracts*** linear, 5–10 mm, obtuse. ***Lower bracts*** lanceolate to lanceolate-linear, 20–30 mm, acute-attenuate. ***Pedicel*** covered with dense tomentose with stellate hairs up to 2 mm. ***Bracteoles*** present, linear, acute. ***Calyx*** 5–6 mm, lobes linear, acute, dense tomentose with stellate. ***Corolla*** 15–20 mm across, yellow, with pellucid glands, tube up to 2 mm, sparse papillose-ciliated hairs inside, dense tomentose with stellate hairs outside. ***Stamens*** 4–5 or 6 (unstable), 4–6 mm long. ***Filaments*** yellow with yellow-whitish hairs, two anterior glabrous near the apex, three posteriors with hairs up to anthers. ***Anthers*** all reniform. ***Ovary*** ellipsoid, dense tomentose with stellate hairs. ***Style*** up to 7 mm long, filiform, green. ***Stigma*** capitate. ***Capsule*** 8–9 × 2–3 mm, ellipsoid, sparse stellate hairs. ***Seeds*** bothrospermous.

##### Distribution in the Arabian Peninsula.

It is an endemic species to Saudi Arabia, which is known from three locations in Medina province (Jabal Al-Figrah, Jabal Radwa, and Jabal Odks), western Saudi Arabia (Fig. 14).

##### Ecology.

*Verbascum medinecum* grows on rocky slopes, gravelly or sandy wadis, roadsides, and abandoned old gardens at altitudes ranging from 1730 to 1981 m. Associated plants include *Asparagus aphyllus* L., *Helianthemum lippii* (L.) Dum.Cours., *Juniperus turbinata* Guss., *Lepidium draba* L., *Malva parviflora* L., *Nepeta deflersiana* Schweinf. ex Hedge, *Rumex vesicarius* L., *Teucrium capitatum* L., and *Vachellia gerrardii* (Benth.) P.J.H.Hurter.

**Figure 13.**
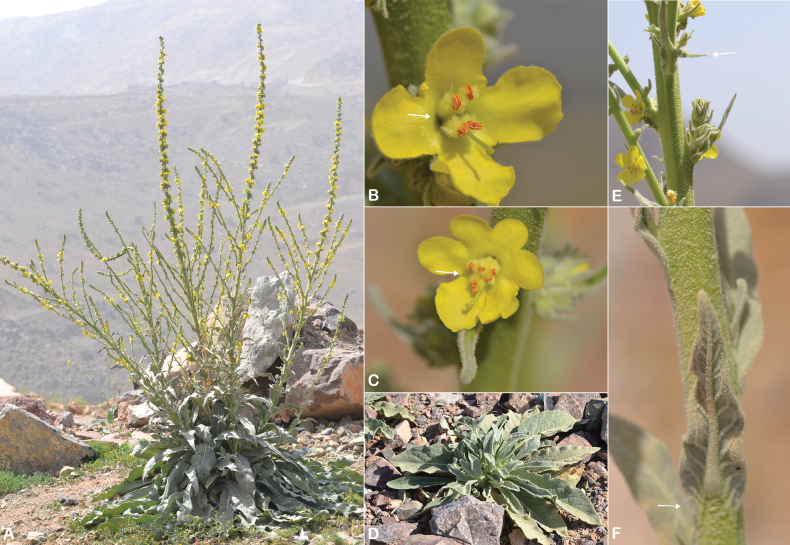
*Verbascum medinecum*. **A.** Habit; **B, C.** Flowers with four and six stamens, respectively; **D.** Leaves; **E.** Upper bracts (white arrow); **F.** Decurrent cauline leaf (white arrow). Photos by Ali Alzahrani.

##### Vernacular name.

*Aithnah*, *Albusira*, *Bousira* (Arabic).

##### Phenology.

Flowering and fruiting from January to August.

##### Etymology.

The name refers to its occurrence in Medina, the holy city in Saudi Arabia.

##### Specimens examined.

**Saudi Arabia.** • **Medina**: Jabal Radwa, 72 km N of Yanbu, 10 February 1987, *I.S. Collenette 5999* (E [E00066950], K, RIY); Home Al-Figrah, 60 km W of Medina, 20 April 1989, *I.S. Collenette 7116* (E [E00066952], K); Home Al-Figrah, 50 km W of Medina, 02 March 1989, *I.S. Collenette 6977* (E [E00066970]); Jabal Radwa, 95 km S of Medina, 31 October 1986, *I.S. Collenette 5889* (E [E00066948]); Jabal Radwa, 70 km N of Yanbu, 10 October 1981, *I.S. Collenette 2899* (E [E00066942]); between Al-Akhal and Umm Al Iyal, new Medina to Jeddah highway, 23 January 1986, *I.S. Collenette 5559* (E [E00066960]); Jabal Al-Figrah, Medina, 07 March 2021, *A. Alzahrani 153* (MUZ).

##### Notes.

Al-Hemaid (2001) described *V. hema-figranum* and *V. medinecum* from Jabal Al-Figrah in the Medina region. Examination of their type specimens, together with phylogenetic results (Alzahrani et al. 2024), indicates that *V. hema-figranum* falls within the variation of *V. medinecum*. Both share: (1) four to five, sometimes six, stamens with yellow-whitish filament hairs; (2) oblong-lanceolate basal leaves; (3) decurrent cauline leaves; (4) lanceolate leaves; and (5) an ellipsoid capsule. Their overlapping distribution in Jabal Al-Figrah further supports this synonymy.

**Figure 14.**
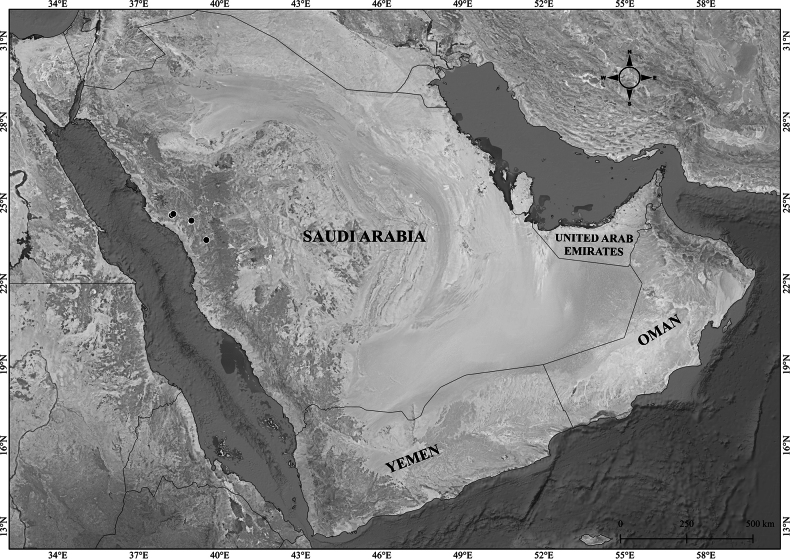
Distribution of *Verbascum medinecum* in the Arabian Peninsula.

#### ﻿8. Verbascum melhanense

Taxon classificationPlantaeLamialesScrophulariaceae

(Murb.) Huber-Morath, Bauhinia 5(1): 14 (1973)

7DBE36F5-A38B-5D5D-9576-86F6D89A6418

Fig. 15

Celsia melhanensis Murb. in Lunds Univ. Arsskrift, n. f. xxii. No.1, 155 (1925).

##### Type.

Yemen, Über Menacha, 23 February 1889, *G. Schweinfurth 1561* (lectotype K designated here).

##### Description.

Biennial herb, dark green to purple, simple or branched from above, up to 90 cm tall. ***Indumentum*** sparse glandular hairs. ***Stems*** erect, terete to angular. ***Basal leaves*** rosette, oblong-ovate, 4–15 × 2–5 cm, apex obtuse, base cordate-truncate, margins crenate-serrate, lamina shiny green with sparse simple hairs on the veins below; petiole 2–6 cm, winged with a few lateral lobes. ***Cauline leaves*** oblong-ovate, 2–6 × 1–3 cm, apex obtuse, base cordate-truncate; sessile or petiole up to 1 cm. ***Inflorescence*** racemose; flowers single in the axil of bracts. ***Upper bracts*** lanceolate or triangular-ovate, 3–5.2 mm, acute. ***Lower bracts*** triangular-ovate, 10–25 mm, acute-attenuate. ***Pedicel*** covered with sparse glandular hairs up to 20 mm long. ***Bracteoles*** absent. ***Calyx*** 4–5 mm, lobes oblong, mucronate, sparse glandular. ***Corolla*** 20–25 mm across, yellow with dark-red streaks on the upper side, without pellucid glands, tube up to 1 mm, sparse papillose hairs inside, sparse glandular hairs outside. ***Stamens*** 4, 10–12 mm long. ***Filaments*** yellow to red with yellowish-violet to red hairs, two anterior glabrous, two shorter posteriors with hairs up to the anthers. ***Anthers*** two anterior inserted decurrent longitudinally on filaments, two shorter posteriors with reniform anthers. ***Ovary*** pyriform-ovoid, sparse glandular hairs. ***Style*** up to 22 mm long, filiform, green. ***Stigma*** capitate. ***Capsule*** 5–8 × 4–6 mm, pyriform-ovoid, sparse glandular hairs. ***Seeds*** bothrospermous.

**Figure 15.**
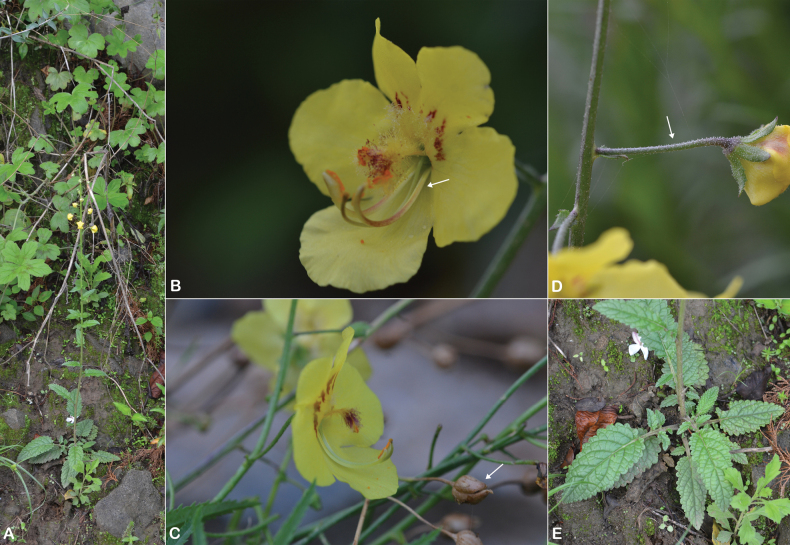
*Verbascum melhanense*. **A.** Habit; **B.** Filaments with two anterior anthers inserted decurrently and longitudinally and glabrous throughout their length (white arrow); **C.** Flowers and capsules (white arrow); **D.** Pedicel with glandular hairs (white arrow); **E.** Leaf. Photos by Ali Alzahrani.

##### Distribution in the Arabian Peninsula.

**Figure 16.**
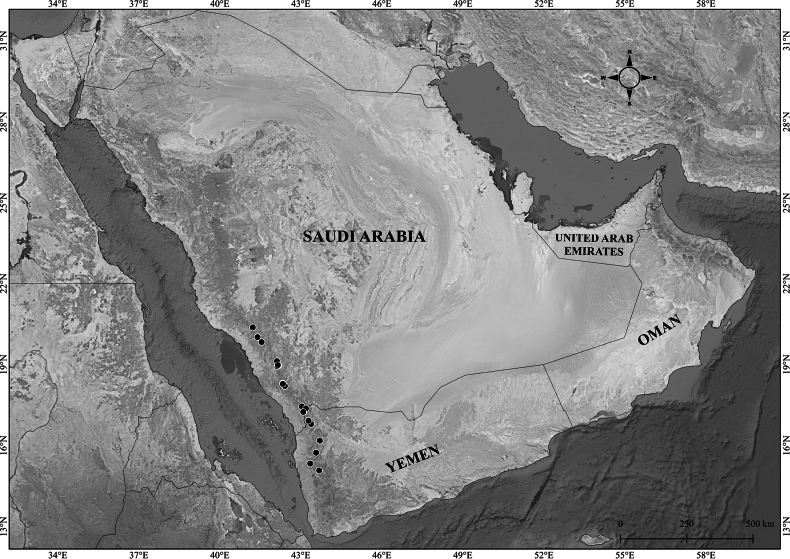
Distribution of *Verbascum melhanense* in the Arabian Peninsula.

It is an endemic species to the Arabian Peninsula, which is known from Saudi Arabia in Al-Baha (King Khalid Road between Qilwah and Al-Baha, Heznah Road between Al-Makhwah and Baljurashi, and Wadi Turbah), Abha (Jabal Mna’a Tanomah, Al-Samma Road, Sinan Road between Al-Namas and Al-Majaradah, Raidah Sanctuary, Near Mahyar Park Tanomah, and Borma’a Road between Tanomah and Bariq), and Jizan (Jabal Fayfa, Jabal Habbes, and Jabal Al-Hasher), and from Yemen in Saada (Jabal Razih and Jabal Marran), Amran (Shaharah), Al-Mahwit (Jabal Melhan), Sana’a (Jabal Masar), and Hajjah (Jabal Nasira), southwestern Arabian Peninsula (Fig. 16).

##### Ecology.

*Verbascum melhanense* grows on mountain cliffs, terrace walls, rocky slopes, granite crevices, and wadi banks at altitudinal ranges from 1600 to 2600 m. Associated plants include *Coleus barbatus* (Andrews) Benth. ex G.Don, *Commelina forskaolii* Vahl, *Crassula schimperi* Fisch. & C.A.Mey., *Cyperus cruentus* Rottb., Dodonaea viscosa subsp. angustifolia (L.f.) J.G.West, *Erigeron bonariensis* L., *Ocimum serpyllifolium* Forssk., *Oxalis corniculata* L., *Pelargonium multibracteatum* Hochst. ex A.Rich., *Selaginella yemensis* (Sw.) Spring, *Solanum incanum* L., and *Withania somnifera* (L.) Dunal.

##### Phenology.

Flowering and fruiting throughout year.

##### Etymology.

The name refers to Jabal Melhan, known locally in Arabic as “Melḥan.”

##### Specimens examined.

**Saudi Arabia.** • **Abha**: Tanomah, about 15 km south of Al-Namas, 10 April 1977, *I.S. Collenette 250* (K); Tanomah, 15 km south of Al-Namas, Abha, 13 April 1979, *I.S. Collenette 1360* (K); between Al-Namas and Tanomah, Tanomah, 07 June 2000, *Taku Miyazaki 000607II34* (E [E00614718]); Aqabat Al-Samma to Wadi Al-Ouse, NW of Abha, 06 January 1981, *A.K. Nasher IH153* (E [E00066915]); Wadi Al-Ouse, Abha, 13 May 1981, *I.S. Collenette 2692* (E [E00066925]); Raidah Sanctuary, Abha, 09 February 2001, *Taku Miyazaki 010209RI* (E [E00614685]); Asir, above Abha, 1946, *W. Thesiger* s.n. (BM); Wadi Al-Ouse, 01 November 1987, *S. Chaudhary 12315* (RIY); Raidah Sanctuary, 21 June 1996, *S. Chaudhary 15523* (RIY); Raidah Sanctuary, 29 April 1998, *T. Al-Turki* & *I.S. Collenette 2610* & *2550* (KSU); Raidah Sanctuary, 21 May 1998, *A. Alfarhan* & *J. Thomas 4840* (KSU); Raidah Sanctuary, 06 July 2007, *A. Alfarhan* & *J. Thomas 6272* (KSU); Raidah Sanctuary, 21 May 1998, *A. Alfarhan* & *J. Thomas 6013* (KSU). • **Jizan**: Jabal Fayfa, about 100 km NE of Jizan, 20 November 1981, *I.S. Collenette 3164* (E [E00066919], K); Jabal Fayfa, 20 November 1981, *S. Chaudhary 998* (RIY); Jabal Fayfa, Jizan, 06 March 1979, *S. Chaudhary 6778* (RIY); Jabal Habbes, near Bani Malik, Jazan, 26 January 2002, *T. Al-Turki* & *J. Thomas 20383* (KSU); Jabal Fayfa, 19 January 1995, *M. Ibrahim 1827* (KSU). • **Al-Baha**: King Khalid Road, NW of Al-Baha, 15 May 1992, *I.S. Collenette 8227* (E [E01000559], K); King Khalid Road between Qilwah and Al-Baha, 13 March 2021, *A. Alzahrani 164* (MUZ).

**Yemen.** • **Sana’a**: Über Menacha, 23 February 1889, *G. Schweinfurth 1561* (K); Jabal Masar, Haraz, 08 June 1979, *J.R.I. Wood 2835* (BM, E [E00066924], K). • **Al-Mahwit**: Jabal Melhan, 15 June 1979, *J.R.I. Wood 2864* (K). **Amran**: Shaharah, 13 November 1981, *J.R.I. Wood 3384* (BM, K). • **Saada**: Jabal Marran, 31 October 1979, *J.R.I. Wood 3036* (K). • **Hajjah**: Jabal Nasira, 08 October 1982, *K. Muller-Hohenstein* & *U. Deil 705* (E [E00066913]).

##### Notes.

*Verbascum melhanense* is morphologically similar to *V. bottae* (see under the latter), but phylogenetic analyses (Alzahrani et al. 2024) support its recognition as a distinct species.

The original material of this species cited by Murbeck (1925) includes the collection *Schweinfurth 1561* from Yemen. Among the available duplicates, the specimen at K is the most complete and best preserved and matches the protologue in all diagnostic characters. To ensure nomenclatural stability, the specimen at K is designated here as the lectotype.

#### ﻿9. Verbascum omanense

Taxon classificationPlantaeLamialesScrophulariaceae

Huber-Morath, Candollea 39(1): 320 (1984)

C52935EA-5F28-51F8-99A5-69D5924569C5

Fig. 17

##### Type.

Oman, Bitinah, Hibra, 10 km N of Nakhl, 2 March 1980, *J.R. Edmondson 3202* (holotype E [E00066934], isotype ON).

##### Description.

Biennial herb, yellowish green, simple, or usually branched from above, up to 1.5 m tall. ***Indumentum*** sparse glandular and stellate hairs above, and dense tomentose with stellate hairs below. ***Stems*** erect, robust, terete. ***Basal leaves*** rosette, oblong to obovate-oblong, 10–20 × 2–6 cm, apex acute or obtuse, base obtuse, margins undulate or lobed-crenate, lamina darkish or yellowish green with sparse stellate hairs above and dense tomentose with stellate hairs below; petiole 1.5–6 cm. ***Cauline leaves*** obovate-oblong, 3–6 × 1–3 cm, apex acute, base cordate; sessile or petiole up to 0.5 cm. ***Inflorescence*** panicle; one or clusters of 2–4 flowers in the axil of bracts. ***Upper bracts*** ovate, 2–3 mm, acute or acuminate. ***Lower bracts*** ovate-triangular to lanceolate-triangular, 10–40 mm, acute or mucronate. ***Pedicel*** covered with sparse glandular and stellate hairs up to 5 mm long. ***Bracteoles*** present, ovate-triangular to lanceolate-triangular, acute. ***Calyx*** 3–4 mm, lobes ovate-oblong, acute, sparse glandular and stellate. ***Corolla*** 15–20 mm across, yellow, with pellucid glands, tubeless, glabrous inside, sparse glandular and stellate hairs outside. ***Stamens*** five, 4–6 mm long. ***Filaments*** orange with violet-whitish hairs, with hairs up to anthers. ***Anthers*** all reniform. ***Ovary*** globose-ovoid, dense tomentose with stellate hairs. ***Style*** up to 7 mm long, filiform, green. ***Stigma*** capitate. ***Capsule*** 4–5 × 2–3 mm, globose-ovoid, dense tomentose with stellate hairs. ***Seeds*** bothrospermous.

##### Distribution in the Arabian Peninsula.

It is an endemic species to the Arabian Peninsula, which is known from Oman in Muscat (Wadi Al Khawd), Al Batinah South (Al Khadra, Hibra, Nakhl, Wadi Abyad, and Wadi Beni Auf), Ash Sharqiyah North (Wadi Dawqah), Ad Dakhiliyah (Jabal Al Halla, Wadi Al Mahil, and Wadi Samail), Al Buraymi (Wadi Rayy), Al Batinah North (Wadi Fizh), and from the United Arab Emirates in Hatta (Wadi Jeema), northeast Oman, and the UAE (Fig. 18).

##### Ecology.

*Verbascum omanense* grows in abandoned or date gardens, roadsides, and rocky wadi beds at altitudes ranging from 100 to 800 m. Associated plants include Dodonaea viscosa subsp. angustifolia (L.f.) J.G.West, *Ficus palmata* Forssk., *Salix acmophylla* Boiss., and *Zygophyllum indicum* (Burm.f.) Christenh. & Byng.

**Figure 17.**
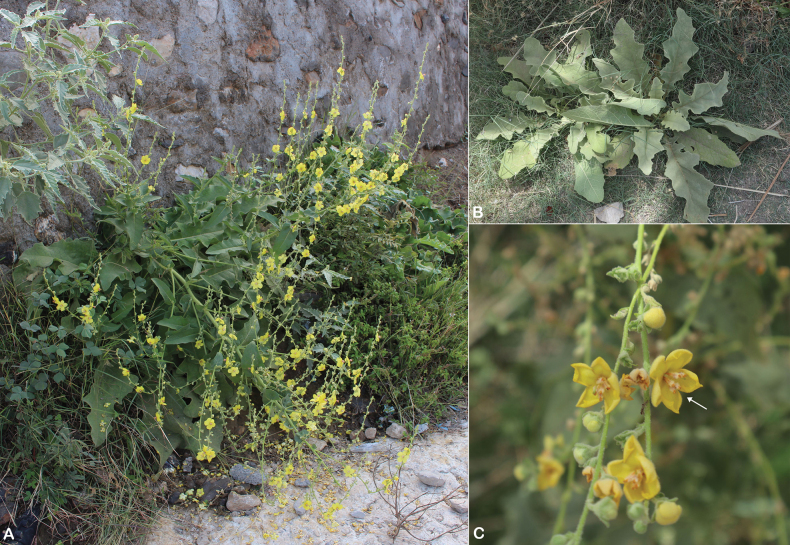
*Verbascum omanense*. **A.** Habit; **B.** Leaf; **C.** Flowers with five stamens (white arrow). Photos: **A.** By Saif Al Hatmi; **B, C.** By Salim Al Rahbi.

##### Phenology.

Flowering and fruiting from February to September.

##### Etymology.

The name refers to its occurrence in Oman.

##### Specimens examined.

**Oman.** • **Al Batinah South**: Al Khadrah, Oasis NE of Buraimi, 23 June 1984, *R.A. Western 655* (E [E00066932]); Hibra, 14 February 1979, *R.P. Whitcombe 472* (E [E00219515], ON); Hibra, near Nakhl, 05 April 1985, *M.D. Gallagher 7487/11* (E [E00066935], ON); Bitinah, Hibra, 10 km N of Nakhl, 02 March 1980, *J.R. Edmondson 3202* (E [E00066934], ON); 1.7 km before Al Thowarah Garden on main road through Nakhl, 23 May 1992, *H.D.V. Prendergast 522* (K, ON); between Al Khod and Nakl, 20 February 2006, *A. Patzelt 2350* (OBG); Wadi Abyad, 24 March 1995, *D. Coshey 274* (ON); Hamiyat al mand, Zammah, Wadi Beni Awf, 20 km S of Rustaq, 11 March 1997, *DHI 278* (ON); Nakhl, 16 March 1995, *D. Coshey 154* (ON); near Wadi Taww to Nakhal Village, 29 June 2021, *A. Alzahrani 191* (MUZ). • **Ash Sharqiyah North**: Northern, Wadi Dawqah, Sharqiyah, 16 April 1993, *I. McLeish 1750* (E [E00128420], ON). • **Ad Dakhiliyah**: W. Hajar mts, 12 km from Kahanat to Rahbah, E. side of Jabal Al Halla, 14 March 1980, *J.R. Edmondson 3399* (E [E00066931]); Wadi Mahil, below Jabal Mahil, S of Sumail, 03 March 1976, *A. Radcliffe-Smith 3766* (K, ON); Wadi Sumail, 03 February 1985, *R.E. Ash 213* (ON); vicinity of Samail, 20 April 1975, *J.P. Mandaville 6694* (BM); vicinity of Samail, 20 April 1975, *J.P. Mandaville 6693* (BM); *Mrs Bovey 69* (BM). • **Al Buraymi**: Wadi Rayy, between Mahdah and Hatta, NE of Buraimi, 04 April 1990, *R.A. Western 1197* (E [E00066933]). • **Al Batinah North**: Wadi Fizh, near Zaymi in mountains NW of Sohar, 08 April 1994, *K.* & *CJN. Roberts & M.D. Gallagher 8598* (E [E00712454], ON). • **Muscat**: Wadi Al Khawd, 14 July 2021, *A. Alzahrani 193* (MUZ).

**Figure 18.**
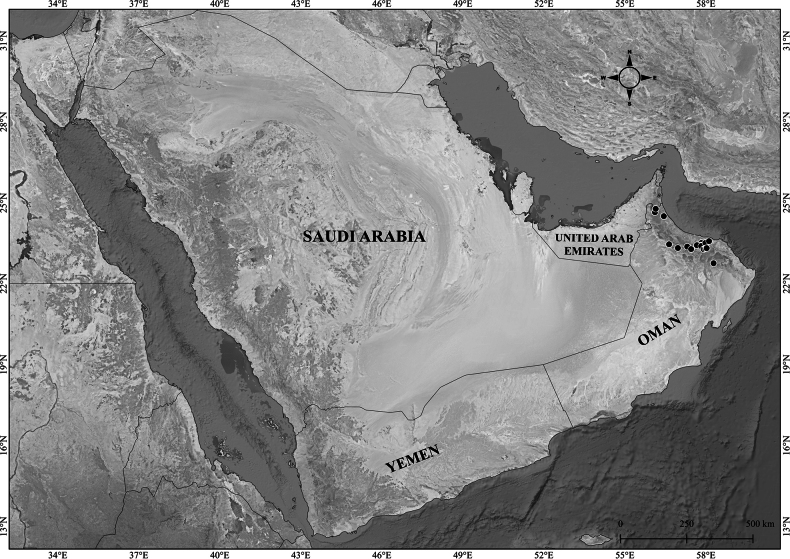
Distribution of *Verbascum omanense* in the Arabian Peninsula.

**United Arab Emirates.** • **Hatta**: Wadi Jeema, Hatta Town, 17 March 1986, *J.N.B. Brown 905* (E [E00066936]).

##### Notes.

*Verbascum omanense* is restricted to the foothills of the Hajar Mountains in Oman and the UAE. It exhibits considerable variation in leaf, bract, and bracteole morphology, sometimes overlapping with *V. sinaiticum*. However, *V. omanense* differs in having glandular hairs, a less dense tomentose indumentum above, a glabrous corolla with glandular-stellate hairs externally, and a globose-ovoid capsule. Phylogenetic analyses (Alzahrani et al. 2024) support its recognition as a distinct species.

#### ﻿10. Verbascum sarawaticum

Taxon classificationPlantaeLamialesScrophulariaceae

A.Alzahrani sp. nov.

FAF17B56-A602-5799-BAD7-B31D4610F30C

urn:lsid:ipni.org:names:77374374-1

Figs 19, 20

##### Remarks.

*Verbascum sarawaticum* resembles *V. yemense* by its indumentum glabrescent or sparse stellate hairs above and dense stellate hairs below, but differs in its growth life biennial (versus perennial), many-branched stems from the base (versus branched from above), basal leaves elliptic-lanceolate (versus oblong to oblong-lanceolate), calyx lobes oblong (versus linear), corolla with pellucid glands (versus without pellucid glands), corolla with sparse ciliated hairs inside (versus glabrous inside), filaments with whitish hairs (versus yellowish hairs), and capsule ovoid (versus ellipsoid-ovoid).

**Figure 19.**
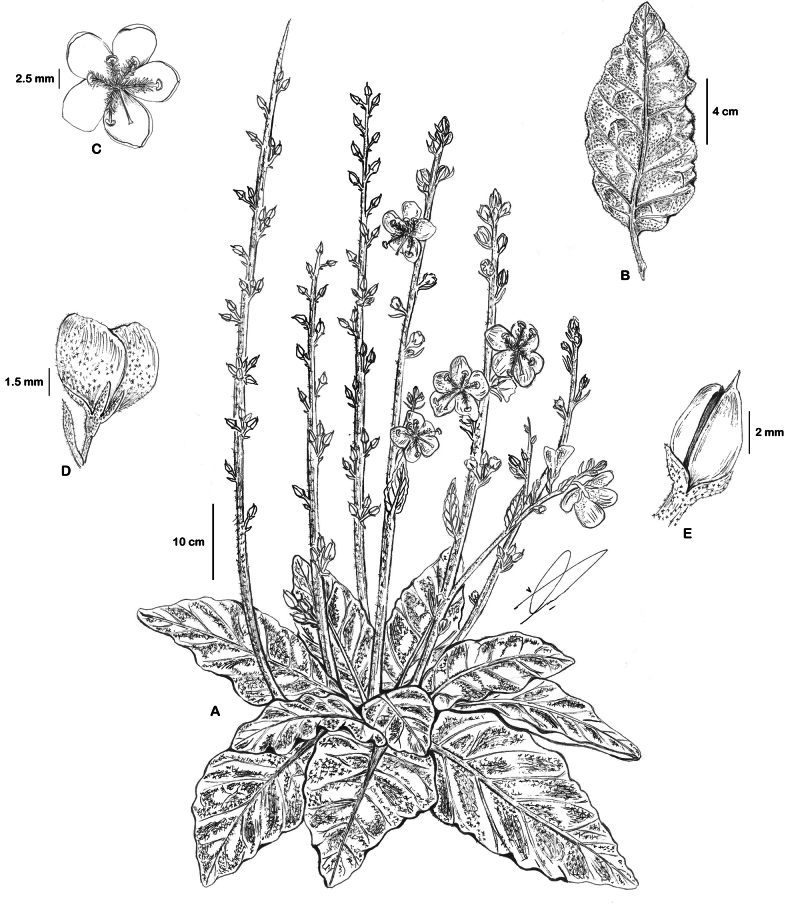
*Verbascum sarawaticum*. **A.** Habit with many-branched stems from the base; **B.** Leaf; **C.** Flower with five stamens, fila ments with two anterior filaments glabrous near the apex; **D.** Flower, calyx, and upper bract covered with sparse stellate hairs; **E.** Capsule. All parts from *I.S. Collenette 2650*. Drawn by Suhair Almalki.

##### Type.

Saudi Arabia, Al-Baha, Red Mountain, 50 km S of Baljurashi, 10 May 1981, *I.S. Collenette 2650* (holotype K).

**Figure 20.**
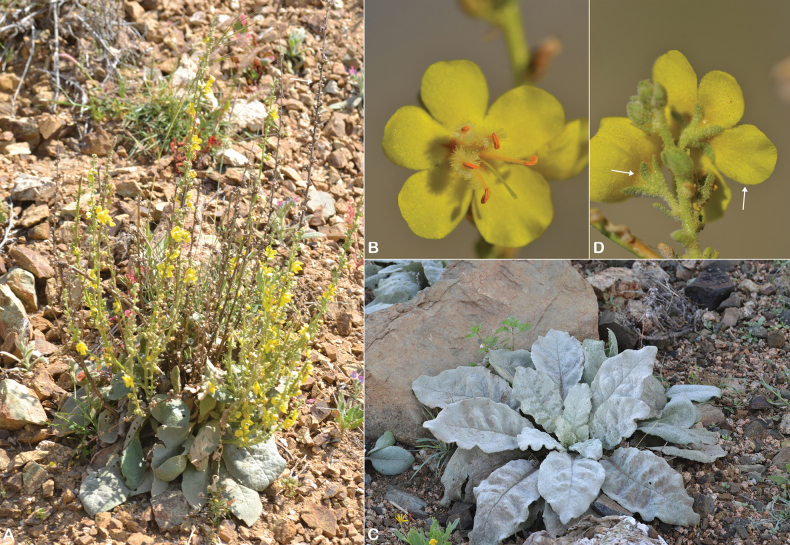
*Verbascum sarawaticum*. **A.** Habit and capsule (white arrow); **B.** Flowers with five stamens and filaments with whitish hairs; **C.** Leaf; **D.** Sparse stellate hairs on the outer surface of the calyx and corolla (white arrows). Photos by Ali Alzahrani.

##### Description.

Biennial herb, yellowish green, simple, or very branched from the base, up to 1 m tall. ***Indumentum*** glabrescent or sparse stellate hairs above and dense stellate hairs below. ***Stems*** erect, terete. ***Basal leaves*** rosette, elliptic-lanceolate, 2–15 × 1.5–6 cm, apex obtuse, base obtuse or cuneate, margins crenate-repand, lamina whitish green with dense stellate hairs; petiole 2–5 cm. ***Cauline leaves*** lanceolate, 1.5–2 × 0.5–1 cm, apex acute, base cuneate; sessile. ***Inflorescence*** racemose forming panicle; one or clusters of 2–3 flowers in the axil of bracts. ***Upper bracts*** linear, 2–3 mm, mucronate. ***Lower bracts*** lanceolate, 10–15 mm, acute. ***Pedicel*** glabrescent or sparse stellate hairs up to 3 mm long. ***Bracteoles*** absent. ***Calyx*** 3–4 mm, lobes oblong, mucronate, sparse stellate hairs. ***Corolla*** 15–20 mm across, yellow with red marks in the throat, with pellucid glands, tube up to 1 mm, sparse ciliated hairs inside, sparse stellate hairs outside. ***Stamens*** five, 3–5 mm long. ***Filaments*** orange with whitish hairs, two anterior glabrous near the apex, three posteriors with hairs up to anthers. ***Anthers*** all reniform. ***Ovary*** ovoid, dense stellate hairs. ***Style*** up to 7 mm long, filiform, green. ***Stigma*** spatulate. ***Capsule*** 4–6 × 2–4 mm, ovoid, sparse stellate hairs. ***Seeds*** 0.7–0.8 × 0.4–0.5 mm, brownish, oblong, bothrospermous.

##### Distribution in the Arabian Peninsula.

**Figure 21.**
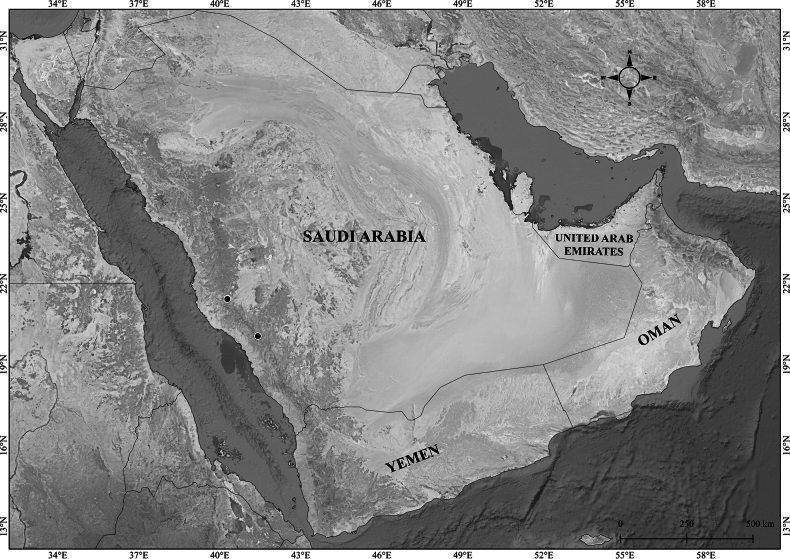
Distribution of *Verbascum sarawaticum* in the Arabian Peninsula.

It is an endemic species to Saudi Arabia, which is known from Al-Baha (Red Mountain in Baljurashi) and Taif (near Al-Hada palm, Al-Hada), southwestern Saudi Arabia (Fig. 21).

##### Ecology.

*Verbascum sarawaticum* grows on granite rubbles and roadsides at altitudinal ranges from 1600 to 1980 m. Associated plants include *Aizoon canariense* L., *Blepharis edulis* (Forssk.) Pers., *Campanula edulis* Forssk., *Commicarpus grandiflorus* (A.Rich.) Standl., *Fumaria abyssinica* Hammar, *Juniperus procera* Hochst. ex Endl., *Osteospermum vaillantii* (Decne.) Norl., *Rumex vesicarius* L., and *Solanum incanum* L.

##### Phenology.

Flowering and fruiting from March to August.

##### Etymology.

The name refers to the Sarawat Mountains, known locally in Arabic as “Sarawat.”

##### Specimens examined.

**Saudi Arabia.** • **Al-Baha**: Red Mountain, 50 km S of Baljurashi, 10 May 1981, *I.S. Collenette 2650* (holo. K). • **Taif**: Al-Hada, 22 March 2005, *A. Alfarhan, T. Al-Turki* & *J. Thomas 4610* (KSU); near Al-Hada palm, Al-Hada, 10 March 2021, *A. Alzahrani 155* (MUZ).

##### Notes.

Phylogenetic analyses (Alzahrani et al. 2024) confirm that *Verbascum sarawaticum* is distinct from the morphologically similar *V. yemense*.

#### ﻿11. Verbascum saudiarabicum

Taxon classificationPlantaeLamialesScrophulariaceae

(A.Alzahrani) A.Alzahrani comb. nov.

C73E2F21-3058-5887-B719-6D2CAF006C94

urn:lsid:ipni.org:names:77374375-1

Rhabdotosperma saudiarabicum A.Alzahrani, Kew Bull. 77(4): 987 (2022).

##### Type.

Saudi Arabia, Abha, Jabal Al-Soudah, Al-Soudah, 25 km NW of Abha, 22 February 1982, *I.S. Collenette 3316* (holotype K).

##### Notes.

Alzahrani et al. (2022) described *Rhabdotosperma saudiarabicum* as a new species from Saudi Arabia; however, recent phylogenetic research (Alzahrani et al. 2024) confirms the inclusion of *Rhabdotosperma* within *Verbascum*. For a detailed description, see Alzahrani et al. (2022).

#### ﻿12. Verbascum schimperianum

Taxon classificationPlantaeLamialesScrophulariaceae

Boiss. Diagn. Pl. Orient. ser. 1, 12: 11 (1853)

F623302E-F092-5977-BD7F-1C4DCDEB1D71

Fig. 22

Verbascum crispum Ehrenb. ex Boiss., Fl. Orient. 4(2): 341 (1879).

##### Type.

Inter Tor et Sinam, *Ehrenberg 300* (lectotype K designated here, isolectotype P [P03285813]).

##### Description.

Perennial herb, yellowish green, very branched from the base, woody at the base, up to 80 cm tall. ***Indumentum*** dense rough yellowish tomentose with stellate hairs. ***Stems*** erect, terete. ***Basal leaves*** rosette, oblong to obovate-oblong, 3–10 × 2–5 cm, apex obtuse, base obtuse, margins sinuate to lobed-crenate, lamina yellowish green with dense yellow-grey tomentose with stellate hairs; petiole 0.5–2 cm. ***Cauline leaves*** oblong-ovate, 3–5 × 1–2 cm, apex obtuse, base cordate, sessile or petiole up to 1 cm. ***Inflorescence*** racemose; flowers single in the axil of bracts. ***Upper bracts*** ovate, 2–3 mm long, acute. ***Lower bracts*** ovate-triangular, 20–50 mm long, acute. ***Pedicel*** covered with dense tomentose with stellate hairs up to 3 mm long. ***Bracteoles*** absent. ***Calyx*** 3–4 mm, lobes ovate-elliptic, acute, dense tomentose with stellate. ***Corolla*** 15–20 mm across, yellow, with pellucid glands, tubeless, sparse ciliated hairs inside, dense tomentose with stellate hairs outside. ***Stamens*** five, 3–6 mm long. ***Filaments*** yellow with yellow hairs, two anterior glabrous near the apex, three posteriors with hairs up to anthers. ***Anthers*** all reniform. ***Ovary*** ellipsoid-ovoid, dense tomentose with stellate hairs. ***Style*** up to 6 mm long, filiform, green. ***Stigma*** capitate. ***Capsule*** 4–5 × 2–3 mm, ellipsoid-ovoid, dense tomentose with stellate hairs. ***Seeds*** bothrospermous.

##### Distribution.

Jordan, Palestine, Egypt (Sinai), and Saudi Arabia.

##### Distribution in the Arabian Peninsula.

It is a native species to Saudi Arabia, which is known from two locations in Tabuk province (Wadi Sawawin, Ain Al-Shayatei, and surrounding areas), northwest Saudi Arabia (Fig. 23).

##### Ecology.

*Verbascum schimperianum* grows among rocks in wadi edges and granite sand in lava at altitudes ranging from 600 to 1280 m. Associated plants include *Haloxylon salicornicum* (Moq.) Bunge ex Boiss., *Ochradenus baccatus* Delile, *Retama raetam* (Forssk.) Webb & Berthel., *Scrophularia deserti* Delile, *Stachys aegyptiaca* Pers., *Zilla spinosa* (L.) Prantl., and *Zygophyllum molle* (Delile) Christenh. & Byng.

##### Phenology.

Flowering from March to November.

##### Etymology.

The name commemorates Georg Wilhelm Schimper (1804–1878), a German botanist and plant collector.

##### Specimens examined.

**Saudi Arabia.** • **Tabuk**: Wadi Sawawin, 26 March 1978, *I.S. Collenette 527* (K).

##### Notes.

*Verbascum schimperianum* is closely related to *V. eremobium* in sharing a dense, rough tomentum composed of stellate hairs, branched stems with a woody base, five stamens, and reniform anthers. It differs, however, in having a racemose inflorescence lacking bracteoles, ovate-elliptic calyx lobes, yellow filaments with yellow hairs, and an ellipsoid-ovoid capsule.

**Figure 22.**
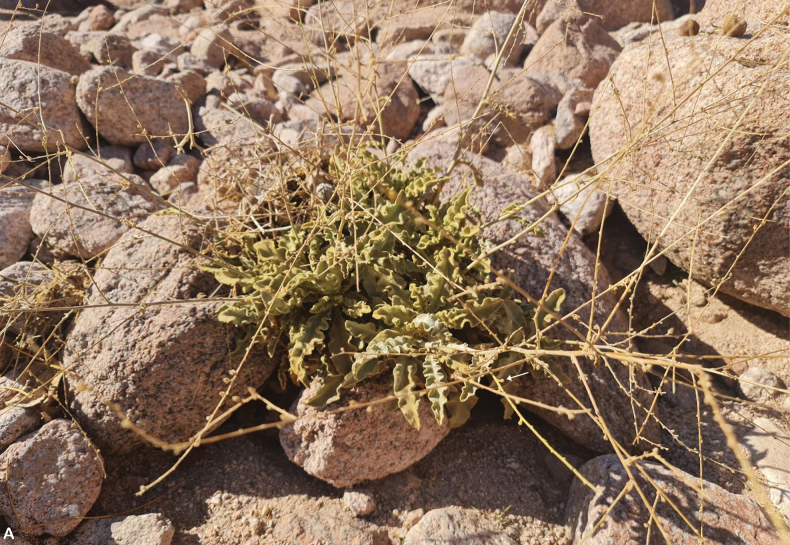
*Verbascum schimperianum*. **A.** Habit and capsule (white arrow). Photos by Abdul Wali Alkhulaidi.

**Figure 23.**
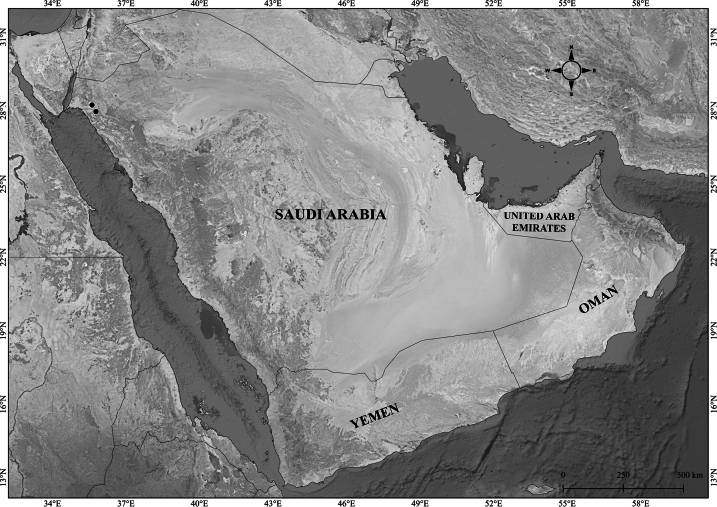
Distribution of *Verbascum schimperianum* in the Arabian Peninsula.

The original material of *Verbascum schimperianum* includes more than one collection attributable to Ehrenberg. To ensure nomenclatural stability, the specimen *Ehrenberg 300* at K is designated here as the lectotype because it best matches the protologue, is complete and well preserved, and represents the most reliable and informative element of the original material. The duplicate at P is treated as an isolectotype.

#### ﻿13. Verbascum shiqricum

Taxon classificationPlantaeLamialesScrophulariaceae

Hemaid, Pakistan J. Bot. 33(4): 324 (2001)

E3419604-1FC9-5FE3-B471-9B507884D583

Fig. 24

Verbascum abyadicum Hemaid, Pakistan J. Bot. 33(4): 316 (2001), syn. nov. – Type: Saudi Arabia, Harrat Khaybar, 125 Km N of Medina, 10 August 1982, *I.S. Collenette 3757* (holotype E [E00066949], isotype K).

##### Type.

Saudi Arabia, Tabuk, between Sawawin and Shiqri, 12 April 1985, *I.S. Collenette 5277* (holotype E [E00066964]).

##### Description.

Biennial herb, yellowish green, simple, or usually branched from the base, up to 1 m tall. ***Indumentum*** glabrescent or sparse stellate hairs above and dense tomentose with stellate hairs below. ***Stems*** erect, robust, terete. ***Basal leaves*** rosette, obovate-elliptic to ovate, 5–15 × 2–10 cm, apex acute, base obtuse to cuneate, margins crenate-sinuate, lamina white or grey greenish with dense white-grey tomentose with stellate hairs; petiole 2–10 cm. ***Cauline leaves*** lanceolate, 3–5 × 1.5–2 cm, apex acuminate, base obtuse-cuneate, sessile or petiole up to 2 cm. ***Inflorescence*** racemose forming panicle; one or clusters of 2–6 flowers in the axil of bracts. ***Upper bracts*** linear, 5–10 mm, acute. ***Lower bracts*** oblong-elliptic, 15–20 mm, acuminate. ***Pedicel*** glabrescent or sparse stellate hairs up to 3 mm long. ***Bracteoles*** absent. ***Calyx*** 5–6 mm, lobes linear, acute, glabrescent, or sparse stellate. ***Corolla*** 15–20 mm across, yellow with red marks in the throat, with pellucid glands, tube up to 2 mm, sparse ciliated hairs inside, sparse stellate hairs outside. ***Stamens*** five, 4–5 mm long. ***Filaments*** orange with whitish hairs, two anterior glabrous near the apex, three posteriors with hairs up to the anthers. ***Anthers*** all reniform. ***Ovary*** ellipsoid, dense tomentose with stellate hairs. ***Style*** up to 8 mm long, filiform, green. ***Stigma*** capitate. ***Capsule*** 5–6 × 3–4 mm, ellipsoid, dense tomentose with stellate hairs. ***Seeds*** bothrospermous.

##### Distribution in the Arabian Peninsula.

It is an endemic species to Saudi Arabia, which is known from several locations in Tabuk province (Alaqan near the Jordan borders, between Duba and Shiqri near Shiqri, and Jabal Qaraqir) and in Medina province (Harrat Khaybar near Jabal Abyad, Al-Ula near Bir Al-Qurr, and Harrat Uwayrid), northwest to western Saudi Arabia (Fig. 25).

##### Ecology.

*Verbascum shiqricum* grows in crevices of black lava, roadsides, and rocky sandstone at altitudes ranging from 915 to 1680 m. Associated plants include *Euphorbia dracunculoides* Lam., *Forsskaolea tenacissima* L., *Matthiola longipetala* (Vent.) DC., *Nanorrhinum acerbianum* (Boiss.) Betsche, *Ononis natrix* L., *Pseudodictamnus undulatus* (Benth.) Salmaki & Siadati, *Rumex vesicarius* L., *Solenostemma arghel* (Delile) Hayne, and *Vachellia gerrardii* (Benth.) P.J.H.Hurter.

**Figure 24.**
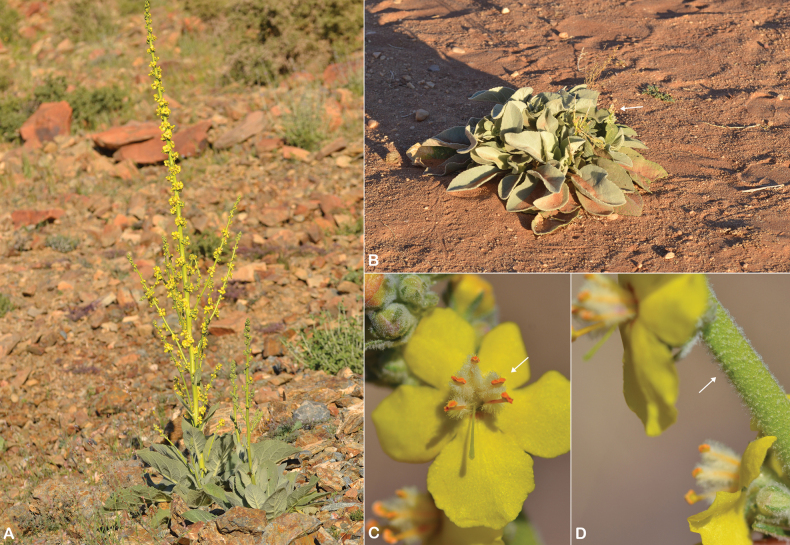
*Verbascum shiqricum*. **A.** Habit; **B.** Leaves, stems grazed (white arrow); **C.** Flowers with five stamens and filaments with whitish hairs (white arrow); **D.** Stems with sparse stellate hairs (white arrow). Photos by Ali Alzahrani.

##### Phenology.

Flowering from March to August.

##### Etymology.

The name refers to the type locality, Shiqri.

##### Specimens examined.

**Saudi Arabia.** • **Tabuk**: between Sawawin and Shiqri, 12 April 1985, *I.S. Collenette 5277* (E [E00066964]); north Hijaz, Wadi Qaraqir, 10 March 1979, *I.S. Collenette 1013* (K); Jabal Hisma ranges, 26 March 1989, *I.S. Collenette 7028* (E [E00066957]); Tabuk road between Duba and Shigry, near Shigry, 16 June 2021, *A. Alzahrani 180* (MUZ). • **Medina**: Harrat Khaybar, 125 Km N of Medina, 10 August 1982, *I.S. Collenette 3757* (E [E00066949], K); near Bir Al-Qurr, Al-Ula, 05 March 2021, *A. Alzahrani 149* (MUZ); Harrat Khaybar, near Jabal Abyad, 06 March 2021, *A. Alzahrani 150* (MUZ).

##### Notes.

*Verbascum shiqricum* is a morphologically distinctive species in Saudi Arabia. Phylogenetic analyses (Alzahrani et al. 2024) also support its recognition as a separate taxon. Detailed examination of the type specimens of *V. abyadicum* and *V. shiqricum*, together with comparison of their key morphological characters, shows that the two names refer to the same species. Both share: (1) glabrescent or sparsely stellate hairs on the upper leaf surface and dense tomentose stellate hairs below; (2) basal leaves obovate-elliptic to ovate; (3) five stamens; (4) racemose inflorescences forming a panicle with solitary flowers or clusters of 2–6 flowers; (5) whitish hairs on the filaments; and (6) an ellipsoid capsule. *Verbascum abyadicum* is therefore treated here as a synonym of *V. shiqricum*.

**Figure 25.**
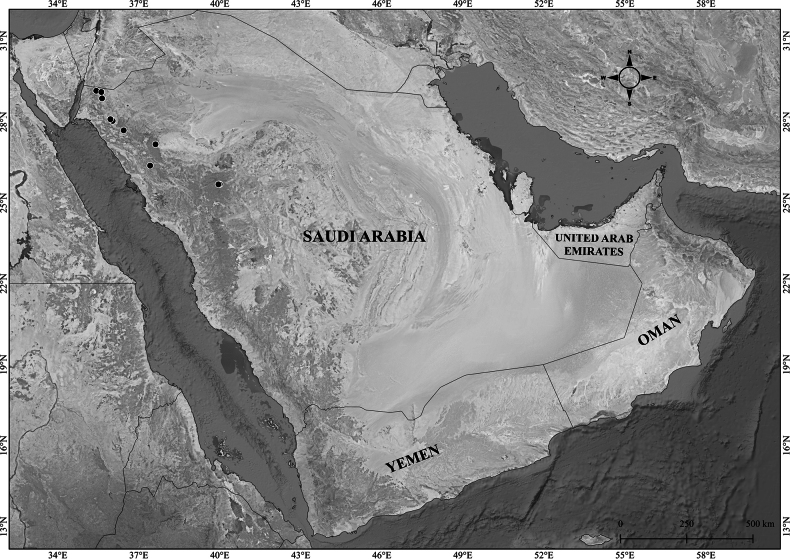
Distribution of *Verbascum shiqricum* in the Arabian Peninsula.

#### ﻿14. Verbascum sinaiticum

Taxon classificationPlantaeLamialesScrophulariaceae

Benth. in DC., Prodr. 10: 236 (1846)

98E28A82-364F-53BC-8274-B3565139809C

Fig. 26

Verbascum fasciculatum Ehrenb. ex Sweet, Hort. Brit., ed. II. p. 381 (1830). – Type: Egypt, Mount Sinai 1829, s.n. (not seen).
Verbascum ternacha Hochst. In: A. Rich. Tent. Fl. Abyss. II: 108. (1851). – Type: Ethiopia, prope Dscheladscheranne 1852, *W. Schimper 621* (isotype M [M0106186]).
Verbascum barradense Boiss., Fl. Orient. 4(2): 318 (1879). – Type: Syria, Damascus, prés au bord du Barrada, gorge de Doumar, 20 June 1853, *C. Gaillardot* s.n. (Hskn.?, not seen).
Verbascum somaliense Baker, Bull. Misc. Inform. Kew (105): 222 (1895). – Type: Somalia, Golis range, Balamha, *E. Cole* & *E. Lort Phillips 296* (lectotype K [K000411058] designated here).
Verbascum nubicum Murb., in Lunds Univ. Arsskrift, n. f. xxix. No. 2. 293 (1933). – Type: Red Sea Hills, Erkowit, *L. Maffey 5* (lectotype K [K000411062] designated here).

##### Type.

Egypt, ad fontes montis Sinai Peninsula, 30 May 1835, *W. Schimper 357* (lectotype HBG [HBG512118] designated here, isolectotype HBG [HBG512120]).

##### Description.

Biennial herb, yellowish green, usually simple large or branched above, up to 2 m tall. ***Indumentum*** dense rough tomentose with stellate hairs. ***Stems*** erect, robust, terete. ***Basal leaves*** rosette, oblong to oblong-ovate, 10–30 × 3–10 cm, apex acute, base obtuse to cuneate, margins crenate-dentate, lamina yellowish green with dense yellow-grey tomentose with stellate hairs; petiole 2–13 cm. ***Cauline leaves*** oblong-ovate, 4–10 × 3–5 cm, apex acute-acuminate, base obtuse; sessile or petiole up to 1 cm. ***Inflorescence*** panicle; clusters of several flowers in the axil of bracts. ***Upper bracts*** ovate, 5–8 mm, acute or acuminate. ***Lower bracts*** ovate to ovate-triangular, 15–40 mm, acuminate. ***Pedicel*** covered with dense tomentose with stellate hairs up to 8 mm long. ***Bracteoles*** present, ovate to ovate-lanceolate, acute. ***Calyx*** 4–5 mm, lobes lanceolate, acute, dense tomentose with stellate. ***Corolla*** 15–20 mm across, yellow, with pellucid glands, tube up to 2 mm, glabrous or sparse ciliated hairs inside, dense tomentose with stellate hairs outside. ***Stamens*** 4–5 or 7 (unstable), 4–6 mm long. ***Filaments*** orange with violet-whitish hairs, with hairs up to anthers. ***Anthers*** all reniform. ***Ovary*** ellipsoid-ovoid, dense tomentose with stellate hairs. ***Style*** up to 8 mm long, filiform, green. ***Stigma*** capitate. ***Capsule*** 4.5–7.5 × 4–6 mm, ellipsoid-ovoid to globose, dense tomentose with stellate hairs. ***Seeds*** bothrospermous.

**Figure 26.**
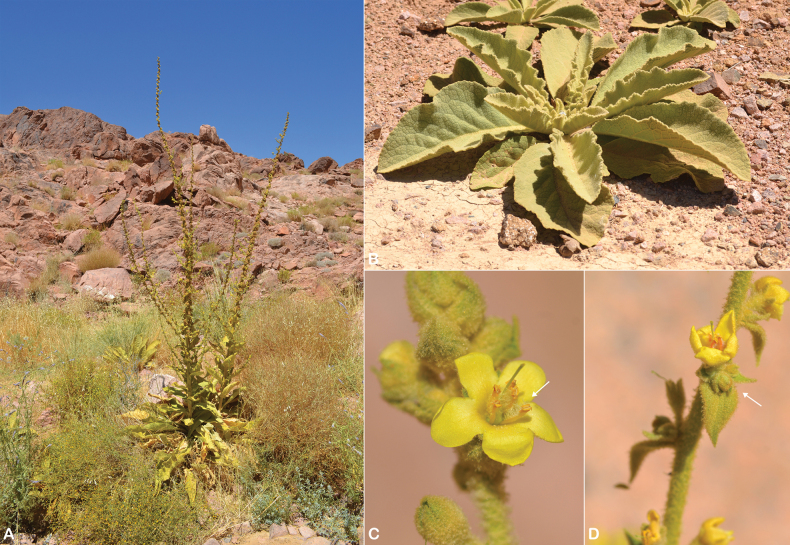
*Verbascum sinaiticum*. **A.** Habit; **B.** Leaf; **C.** Flowers with five stamens and filaments with hairs up to the anthers (white arrow); **D.** Calyx and upper bracts (white arrow). Photos by Ali Alzahrani.

##### Distribution.

Eritrea, Ethiopia, Kenya, Somalia, Sudan, Niger, Egypt, Iraq, Jordan, Lebanon, Syria, Palestine, and the Arabian Peninsula.

##### Distribution in the Arabian Peninsula.

**Figure 27.**
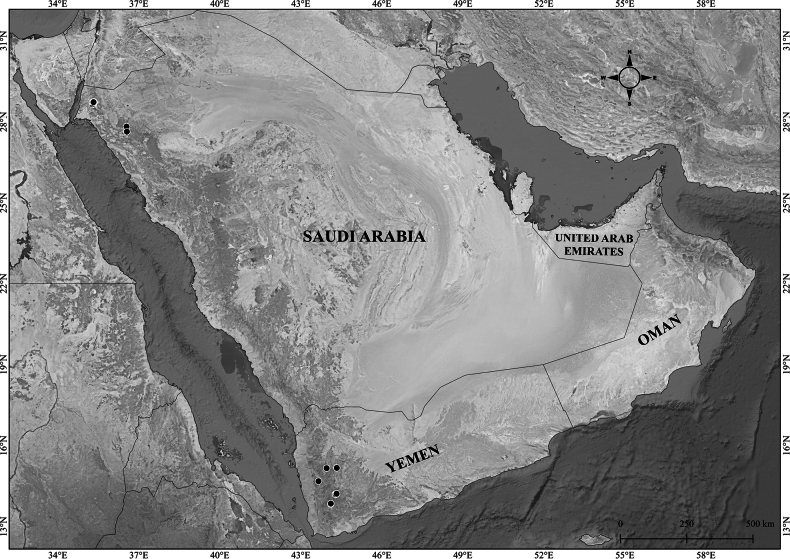
Distribution of *Verbascum sinaiticum* in the Arabian Peninsula.

It is a native species to Saudi Arabia, which is known from Tabuk province (Jabal Al-Lawz, Wadi Al-Disah, Harrat Raha) northwest Saudi Arabia, but it is an introduced species from Yemen, where it is known from Sana’a (Haima Al Kharajia and between Sayyan and Qaidun), Raymah (Jibal Raymah), and Ibb (near Dhi Sufal and Sumara Pass) southwestern Yemen (Fig. 27).

##### Ecology.

*Verbascum sinaiticum* grows on rocky slopes or granite, hillsides, beds of sandy wadis, black lava, and roadsides at altitudes ranging from 1500 to 2800 m. Associated plants include *Dianthus sinaicus* Boiss., *Hypericum sinaicum* Hochst. ex Boiss., *Kickxia collenetteana* D.A.Sutton, *Lactuca orientalis* (Boiss.) Boiss., *Nanorrhinum acerbianum* (Boiss.) Betsche, *Ononis natrix* L., *Phlomis brachyodon* (Boiss.) Zohary ex Rech.f., *Pistacia khinjuk* Stocks, *Pterocephalus sanctus* Decne., and *Verbascum decaisneanum* O. Kuntze.

##### Vernacular name.

*Qetetina* (English); *Aithnah*, *Albusira* (Arabic).

##### Phenology.

Flowering and fruiting from May to August.

##### Etymology.

The name refers to its occurrence in Sinai, Egypt.

##### Specimens examined.

**Egypt.** • **Sinai Peninsula**: ad fontes montis Sinai, 30 May 1835, *W. Schimper* 357 (HBG [HBG512118] & [HBG512120]).

**Saudi Arabia.** • **Tabuk**: Jabal Al-Lawz, near Aqaba Gulf, 03 August 1989, *I.S. Collenette 7235* (E [E00066927], K); Harrat Raha, 20 km SE of Badiah, 17 May 1994, *I.S. Collenette 9144* (E [E00092224], K); Jabal Al-Lawz, S of Aqaba, Wadi Lakus, 05 May 1992, *I.S. Collenette 8213* (K); Jabal Al-Lawz, N of Tabuk, 02 June 2016, *J. Thomas*, *M. El-Sheikh* & *A. Alatar 24311* (KSU); Wadi Al-Disah, SW of Tabuk, 01 April 2014, *J. Thomas 23741* (KSU); Jabal Al-Lawz, Tabuk, 06 May 2016, *M. El-Sheikh* & *M. Al-Shehri 23366* (KSU); Jabal Al-Lawz, 17 June 2021, *A. Alzahrani 181* (MUZ).

**Yemen.** • **Sana’a**: Haima Al Kharajia, Manakhah to Sana’a, 29 November 1976, *J.R.I. Wood 1483* (K); between Sayyan and Qaidun, 01 May 1977, *J.R.I. Wood 1579* (BM, K). • **Raymah**: Jibal Raymah, path below ridge to S of Al Jabin, 21 March 1984, *A. G. Miller* & *R. A. King 5327* (E [E00066966]). • **Ibb**: Near Dhi Sufal, 04 October 1976, *J.R.I. Wood 1351* (E [E00687347]); the road near Ad Delil at the bottom of the Sumara Pass, 05 May 1975, *J.R.I. Wood 75/108* (BM, E [E00066959]); Sumara Pass, 10 km S of Yarim, 01 September 1976, *B. Acres 14* (K); 35 km N of Taiz around Dhi Sufal, 20 October 1975, *F.N. Hepper* & *J.R.I. Wood 5854* (K); Sumara Pass, road summit, 13 October 1975, *F.N. Hepper 5650* (K).

##### Notes.

*Verbascum sinaiticum* is native to northwestern Saudi Arabia but was introduced to Yemen by Rathjens in 1937 (Wood 1997). It differs from *V. shiqricum* in having a dense, rough, tomentose indumentum composed of stellate hairs, a paniculate inflorescence with clusters of several flowers in the bract axils, and an ellipsoid-ovoid to globose capsule. Recent phylogenetic analyses (Alzahrani et al. 2024) support its recognition as a distinct species. Several names historically associated with *Verbascum sinaiticum* were described without designation of a holotype, resulting in multiple original specimens. To stabilize the application of each name, a lectotype was selected from the original material that best conforms to the diagnostic features given in the protologue and is the most complete and well-preserved specimen. For *V. sinaiticum*, the specimen *Schimper 357* at HBG was selected as the lectotype because it most closely matches the protologue and provides a clear interpretation of the name. Similarly, lectotypes were designated for *V. somaliense* and *V. nubicum* from the most representative and complete original collections available.

#### ﻿15. Verbascum transjordanicum

Taxon classificationPlantaeLamialesScrophulariaceae

Murb. in Lunds Univ. Arsskrift, n. f. xxxv. No. 1 54 (1939)

FCBE29A9-3ED5-5C97-A0E0-6F0471D5147E

Fig. 28

##### Type.

Jordan, Sandy desert, 40 km west of Azrak, 17 April 1936, *J.E. Dinsmore 11804* (isotype E [E00327349]).

##### Description.

Annual or biennial herb, pale green, usually branched, many-stemmed from the base, up to 70 cm tall. ***Indumentum*** dense glandular hairs with sparse simple and forked hairs above, and dense tomentose with stellate hairs below. ***Stems*** erect, robust, terete to angular. ***Basal leaves*** rosette, oblong-lanceolate, 2.5–12 × 1–3.5 cm, apex acute, base obtuse, margins crenate to pinnatifid-lobed, lamina darkish green with sparse stellate hairs above and dense white-grey tomentose with stellate hairs below; petiole 1–4 cm. ***Cauline leaves*** oblong-lanceolate, 1.6–5 × 1–2 cm, apex acute, base obtuse; sessile. ***Inflorescence*** racemose; flowers single in the axil of bracts. ***Upper bracts*** ovate-triangular, 1–4 mm, acute. ***Lower bracts*** oblong-lanceolate, 4–8 mm, acute. ***Pedicel*** covered with dense glandular hairs up to 20 mm long. ***Bracteoles*** absent. ***Calyx*** 3.4–4.4 mm, lobes oblong-lanceolate, acute, dense glandular. ***Corolla*** 20–30 mm across, yellow with dark purple-red hairy blotches around the throat, without pellucid glands, tube up to 1 mm, dense papillose hairs inside, spare glandular, simple, and forked hairs outside. ***Stamens*** five, 3–5 mm long. ***Filaments*** yellow with creamy hairs, two anterior glabrous near the apex, three posteriors with hairs up to anthers. ***Anthers*** all reniform. ***Ovary*** globose-ovoid, sparse glandular hairs. ***Style*** up to 7 mm long, filiform, green. ***Stigma*** capitate. ***Capsule*** 4–6 × 3–4 mm, globose-ovoid, sparse glandular hairs. ***Seeds*** bothrospermous.

**Figure 28.**
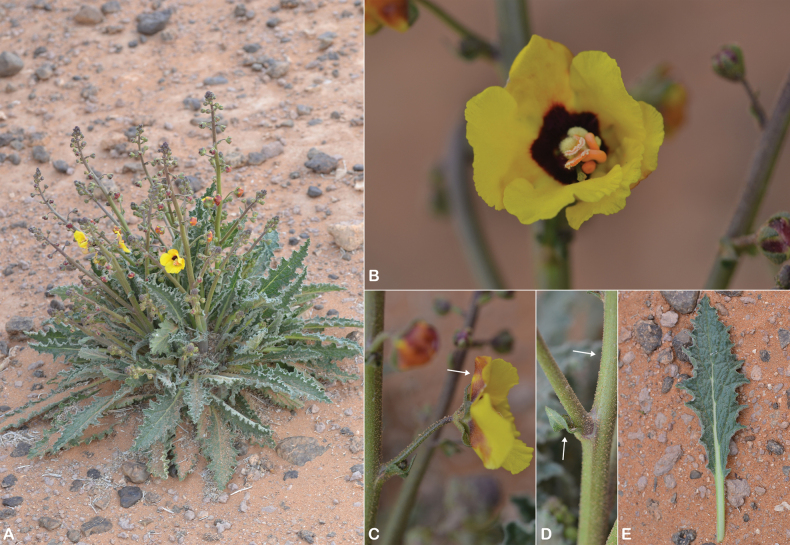
*Verbascum transjordanicum*. **A.** Habit; **B.** Flowers with five stamens and filaments with creamy hairs; **C.** Sparse glandular, simple, and forked hairs on the exterior of the corolla (white arrow); **D.** Stems with subsessile glandular hairs and upper bracts (white arrows); **E.** Leaf. Photos by Ali Alzahrani.

##### Distribution.

Jordan and Saudi Arabia.

##### Distribution in the Arabian Peninsula.

**Figure 29.**
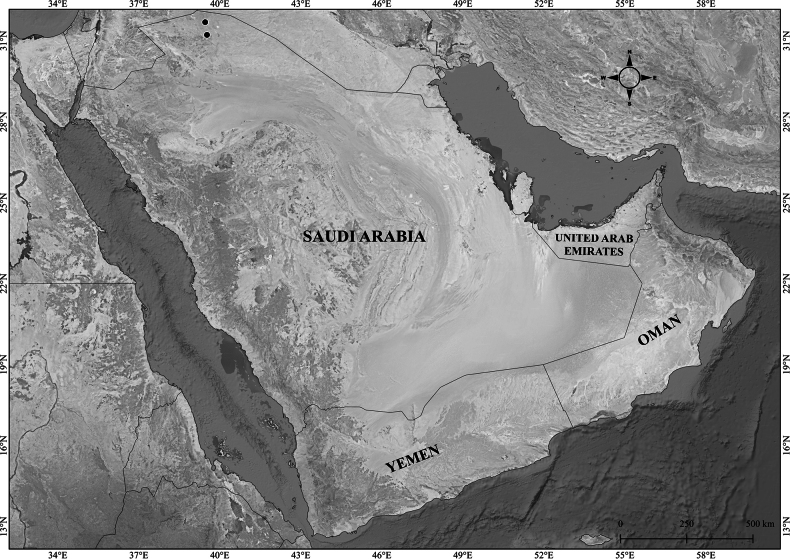
Distribution of *Verbascum transjordanicum* in the Arabian Peninsula.

It is a native species to Saudi Arabia, which is known from Turaif province (near Harrat Al-Harrat Reserve), northern Saudi Arabia (Fig. 29).

##### Ecology.

*Verbascum transjordanicum* grows on a limestone plateau with basalt rock at altitudes ranging from 600 to 832 m. Associated plants include *Achillea fragrantissima* (Forssk.) Sch.Bip., *Centaurea sinaica* DC., *Cornulaca setifera* (DC.) Moq., *Helianthemum lippii* (L.) Dum.Cours., and Hyoscyamus muticus subsp. muticus.

##### Vernacular name.

*Transjordan Mullein* (English), *Abu Ain* (Arabic).

##### Phenology.

Flowering and fruiting from March to June.

##### Etymology.

The name refers to its occurrence in the Transjordan region, meaning “across” or “beyond” Jordan.

##### Specimens examined.

**Jordan.** • **Azrak**: Sandy desert, 40 km west of Azrak, 17 April 1936, *J.E. Dinsmore 11804* (E [E00327349]).

**Saudi Arabia.** • **Turaif**: 5 km NNW of Turaif Camp, 22 April 1994, *I.S. Collenette 9092* (E [E00092227] & [E00092228]); near Harrat Al-Harrat Reserve, 04 March 2021, *A. Alzahrani 148* (MUZ).

##### Notes.

*Verbascum transjordanicum* is distinguished by its solitary flowers, subsessile glandular stems, simple and sparsely forked hairs on the exterior of the corolla, and five stamens, with the two anterior filaments glabrous near the apex and the three posterior filaments bearing creamy hairs up to the anthers. Phylogenetic analyses (Alzahrani et al. 2024) further support its status as a distinct species.

#### ﻿16. Verbascum virgatum

Taxon classificationPlantaeLamialesScrophulariaceae

Stokes, Bot. Arr. Brit. Pl., ed. 2. 1: 227 (-229) (1787)

AF4D9E10-E20B-5EB3-8073-8B76CF9F4ED4

Fig. 30

Blattaria virgata Fourr., Ann. Soc. Linn. Lyon sér. 2, xvii. (1869) 125.
Verbascum blattarioides var. lusitanicum Schrad. in Monogr. Verbasci 2: 45, 47 (1823).
Verbascum virgatum var. lanceolatum Mariz, in Bol. Soc. Brot. 23: 42 (1907)
Verbascum virgatum subsp. lusitanicum (Schrad.) Rivas Goday, in Veg. Fl. Guadiana: 752 (1964).

##### Type.

United Kingdom, Hedge banks, in gravelly soil, in a field on the S. side of the lane leading from Gregory’s Mill to the turnpike road, near that town. The side of the turnpike road from Worcester to Ombersley, opposite to the lane leading to Beverley (not seen).

**Figure 30.**
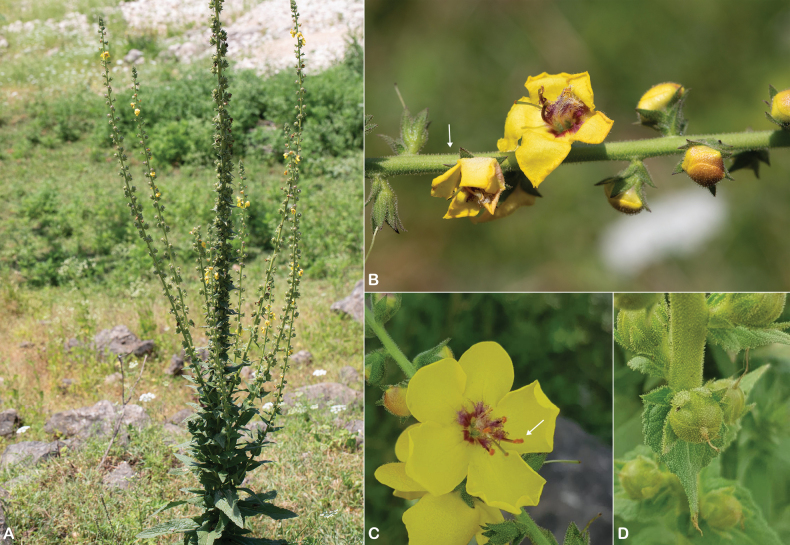
*Verbascum virgatum*. **A.** Habit; **B.** Stems with glandular hairs (white arrow); **C.** Filaments with two anterior anthers inserted obliquely (white arrow); **D.** Capsule. Photos: **A, B.** By Saif Al Hatmi; **C, D.** By Ahmed Jaboob.

##### Description.

Biennial herb, yellowish to reddish green, simple, or branched from above, up to 1.5 m tall. ***Indumentum*** glabrescent or densely glandular hairy. ***Stems*** erect, terete. ***Basal leaves*** rosette, oblanceolate, 8–20 × 3–6 cm, apex acute, base cuneate or attenuate, margins crenate to dentate, lamina darkish green with glabrescent or dense glandular hairs and sparse simple hairs; petiole 1–4 cm. ***Cauline leaves*** oblanceolate, 3–8 × 1–3 cm, apex acute, base cordate; sessile or petiole up to 1 cm. ***Inflorescence*** in spiciform racemes; single flowers or I clusters of 2–3 in the axil of bracts. ***Upper bracts*** triangular-ovate, 3–6 mm, acuminate. ***Lower bracts*** oblanceolate, 15–20 mm, acute to acuminate. ***Pedicel*** glabrescent or dense glandular hairs up to 3 mm long. ***Bracteoles*** present, ovate to oblanceolate, acuminate. ***Calyx*** 4–8 mm, lobes oblanceolate, acute or mucronate, glabrescent or densely glandular hairy. ***Corolla*** 20–25 mm across, yellow with a purple-red spot around the throat, with pellucid glands, tube up to 1 mm, sparse ciliated hairs inside, sparse, or dense glandular hairs outside. ***Stamens*** five, 3–6 mm long. ***Filaments*** red with purple-violet to violet-whitish hairs, two anterior glabrous near the apex, three posteriors with hairs up to anthers. ***Anthers*** two anterior inserted obliquely on filaments, three posteriors with reniform anthers. ***Ovary*** globose, glabrescent, or dense glandular hairs. ***Style*** up to 6 mm long, filiform, green. ***Stigma*** capitate. ***Capsule*** 6–9 × 5–8 mm, globose, glabrescent, or dense glandular hairs. ***Seeds*** bothrospermous.

##### Distribution.

Native to Europe and introduced elsewhere.

##### Distribution in the Arabian Peninsula.

**Figure 31.**
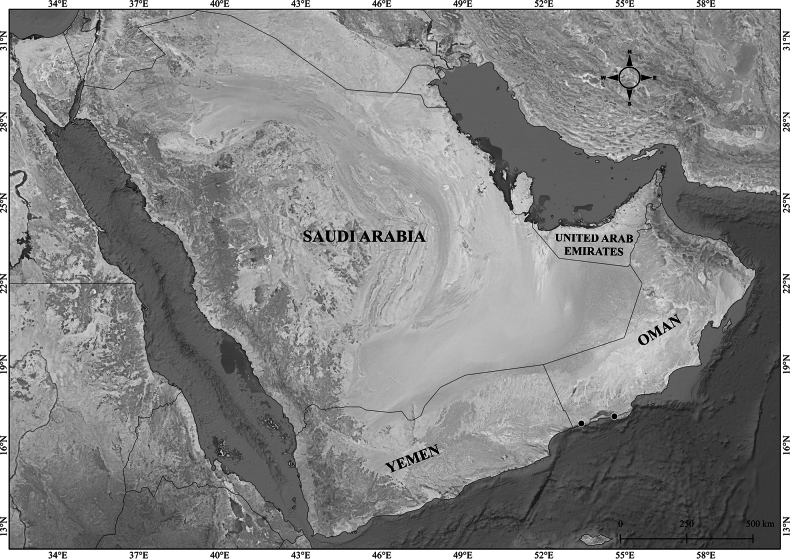
Distribution of *Verbascum virgatum* in the Arabian Peninsula.

It is an introduced species to Oman, which is known from Dhofar (Jabal Qamar) (Fig. 31).

##### Ecology.

*Verbascum virgatum* grows on roadsides and in abandoned gardens at altitudes ranging from 800 to 980 m. There are no records of associated plants.

##### Vernacular name.

*twiggy Mullein* (English).

##### Phenology.

Flowering and fruiting from June to October.

##### Etymology.

The name refers to the species’ twiggy habit, characterized by upright, slender twigs.

##### Specimens examined.

**Oman.** • **Dhofar**: Jabal Qamar, Kezat Amqat, 10 September 2022, *L. Al-Harthy* & *A. Al-Hinai 186* (OBG).

##### Notes.

*Verbascum virgatum* was recently recorded as an introduced species in the Dhofar region of Oman (Al Hatmi et al. 2024).

#### ﻿17. Verbascum yemense

Taxon classificationPlantaeLamialesScrophulariaceae

Defl. Voyage Au Yemen, p.177 (1889)

AC0EFE0E-261D-558B-A604-FE3F2CC91262

Verbascum chaudharyanum Hemaid, Pakistan J. Bot. 33(4): 318 (2001), syn. nov. – Type: Saudi Arabia, An Nimas, Taif-Abha Road, Abha, 28 April 1985, *I.S. Collenette 5321* (holotype E [E00066941], isotype K).

##### Type.

Yemen, ad margines agrorum circa Raudah, 13 June 1887, *A. Deflers 492* (isotype MPU [MPU020119]).

### ﻿Key to the varieties

**Table:** 

1	Pedicels up to 5 mm long	**17a. V. yemense var. yemense**
–	Pedicels up to 15 mm long	**17b. V. yemense var. asiricum**

#### ﻿17a. Verbascum yemense var. yemense

Taxon classificationPlantaeLamialesScrophulariaceae

78568B41-A298-5198-9896-4872314CC574

Fig. 32

##### Description.

Perennial herb, yellowish green, simple, or branched from above, up to 80 cm tall. ***Indumentum*** glabrescent or sparse stellate hairs above and dense stellate hairs below. ***Stems*** erect, terete. ***Basal leaves*** rosette, oblong to oblong-lanceolate, 2–11 × 3–5 cm, apex acute, base obtuse or truncate, margins repand-crenulate, lamina yellowish green with dense stellate hairs; petiole 2–6 cm. ***Cauline leaves*** lanceolate, 1.5–4 × 0.5–1 cm, apex acute-attenuate, base cuneate; sessile or petiole up to 1 cm. ***Inflorescence*** racemose forming panicle; one or clusters of 2–4 flowers in the axil of bracts. ***Upper bracts*** linear, 5–7 mm, acute. ***Lower bracts*** linear-lanceolate, 10–15 mm, acute. ***Pedicel*** glabrescent or sparse stellate hairs up to 5 mm long. ***Bracteoles*** absent. ***Calyx*** 3–4 mm, lobes linear, acute, glabrescent, or sparse stellate. ***Corolla*** 15–20 mm across, yellow, without pellucid glands, tube up to 1 mm, glabrous inside, sparse stellate hairs outside. ***Stamens*** 4–5 or 6 (unstable), 3–5 mm long. ***Filaments*** orange with yellowish hairs, two anterior glabrous near the apex, three posteriors with hairs up to anthers. ***Anthers*** all reniform. ***Ovary*** ellipsoid-ovoid, sparse stellate hairy. ***Style*** up to 6 mm long, filiform, green. ***Stigma*** spatulate. ***Capsule*** 3–4 × 1–3 mm, ellipsoid-ovoid, sparse stellate hairy. ***Seeds*** bothrospermous.

##### Distribution in the Arabian Peninsula.

It is an endemic species to the Arabian Peninsula, which is known from Yemen in Sana’a (Shibam, Jabal An Nabi Shu’ayb, between Sana’a and Walan, Dhamar Road, Beit El-Ghofr, and North Haz), Hajjah (Jabal Jabar), Ibb (Yarim), and Amran (south of Khamr), and from Saudi Arabia in Abha (near Al-Jarrah National Park, north of Alaya, Al-Namas, Tanomah, between Tatlith and Khamis Mushayt, Najran Road, Al-Soudah Road, Al-Soudah, Jabal Mna’a, and King Faisal Road between Bani Amr and Al-Namas), Taif (near Bani Saad, Wadi Thi Ghazal, and Ash Shafa), and Jizan (Jabal Al-Aswad), southwestern the Arabian Peninsula (Fig. 33).

##### Ecology.

*Verbascum yemense* grows on roadsides and abandoned gardens at altitudes ranging from 1800 to 2500 m. Associated plants include *Anthemis yemensis* Podlech, *Erica arborea* L., Felicia abyssinica var. schimperi (Steud. & Hochst. ex Jaub. & Spach) Mesfin, *Lavandula citriodora* A.G.Mill., *Maesa lanceolata* Forssk., *Plantago lanceolata* L., and *Vachellia origena* (Hunde) Kyal. & Boatwr.

##### Phenology.

Flowering and fruiting from March to November.

##### Etymology.

The name refers to the species’ occurrence in Yemen.

##### Specimens examined.

**Saudi Arabia.** • **Abha**: An Nimas, Taif-Abha Road, Abha, 28 April 1985, *I.S. Collenette 5321* (E [E00066941], K); 40–42 km S of Abha, near Al-Jarrah National Park and Tamniah village, 24 May 1980, *L. Boulos* & *A.S. Ads 14259* (K); Talha Camp, old mine at Arjh, 02 April 1974, *I.S. Collenette 231* (K); Asir Mts. just below summit of ridge, 1972, *I.S. Collenette 184* (K); 18 km north of Alaya, 23 April 1990, *I.S. Collenette 7462* (E [E00066961], K); 10 miles east of Hamdah camp, between Tatlith and Khamis Mushayt, 25 March 1977, *I.S. Collenette 53* (K); Al-Namas, 28 April 1985, *I.S. Collenette 5321* (E [E00066941], K); Tanomah, 13 April 1979, *I.S. Collenette 1348* (K); 15 miles east of Hamdah camp, between Tatlith and Khamis Mushayt, 25 March 1977, *I.S. Collenette* 71 (K); 70 km, S.E. of Abha, on road to Najran, 14 March 1980, *J.J. Lavranos* & *I.S. Collenette 18341* (E [E00066937]); 15 km N of Abha, 21 April 1984, *I.S. Collenette 4915* (E [E00066938]); Asir above Dhahran, 12 May 1946, *W. Thesiger* s.n. (BM); Soda, 10 August1952, *J.D. Tothill 147* (BM); Al-Soudah Road, Al-Soudah, 20 March 2021, *A. Alzahrani 177* (MUZ); Jabal Mna’a, Tanomah, 20 March 2021, *A. Alzahrani 179* (MUZ); King Faisal Road between Bani Amr and Al-Namas, 15 March 2021, *A. Alzahrani 172* (MUZ). • **Taif**: Near Ash Shafa, Wadi Thi Ghazal, 20 March 1991, *I.S. Collenette 7716* (E [E00090893], K); near Bani Saad, 11 March 2021, *A. Alzahrani 161* (MUZ); Wadi Thi Ghazal, Ash Shafa, 10 March 2021, *A. Alzahrani 156* (MUZ). • **Jizan**: Jabal Al-Aswad, 13 February 2021, *A. Alzahrani 145* (MUZ).

**Figure 32.**
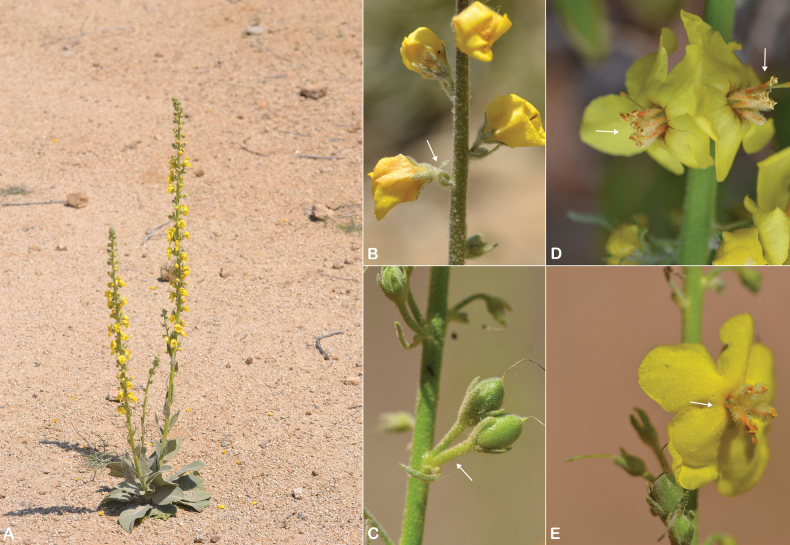
Verbascum yemense var. yemense. **A.** Habit; **B.** Short pedicel; **D.** Flowers with five and six stamens (white arrows). V. yemense var. asiricum. **C.** Long pedicel; **E.** Flowers with five stamens (white arrow). Photos by Ali Alzahrani.

**Yemen.** • **Sana’a**: Shibam, 01 June 1977, *J.R.I Wood 1669* (BM, K); Haddah, 07 November 1975, *F.N. Hepper 6299* (K); Jabal An Nabi Shu’ayb, 20 September 1978, *A.G. Miller 143* (E [E00066954], K); roadside at Kilo 22 between Sana’a and Walan, 14 December 1977, *J.R.I Wood 2158* (BM, K); roadside at Sana’a to Dhamar Road, 10 October 1974, *J.M. Ritchie 62* (E [E00066953]); Jabal An Nabi Shu’ayb, near Yazil, 07 December 1979, *J.R.I Wood 3097* (E [E00066956]); Beit El-Ghofr, North Haz, 04 February 1938, *H. Scott* & *E.B. Britton 494* (BM). • **Hajjah**: Jabal Jabar, 06 June 1946, *W. Thesiger* s.n. (BM). • **Ibb**: Yarim, 27 September 1972, *J.R.I Wood 35* (BM). • **Amran**: 1 mile south of Khamr, 27 August 1975, *J.R.I Wood 75/600* (BM).

**Figure 33.**
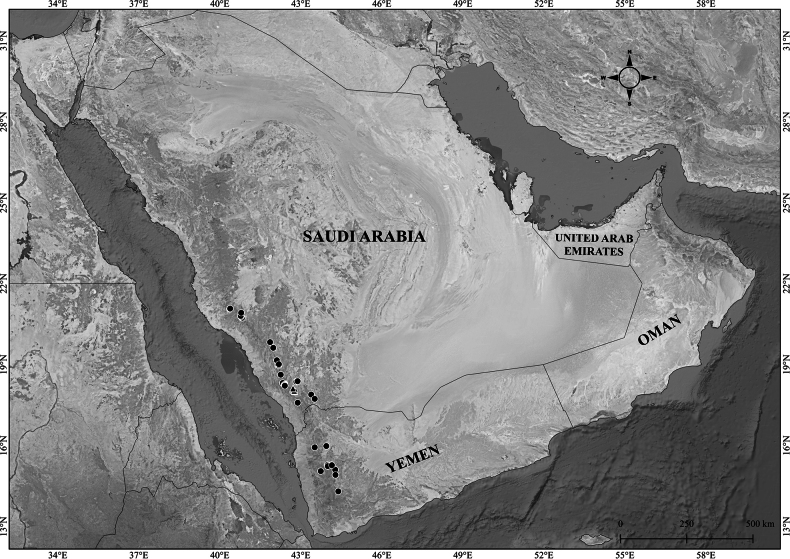
Distribution of Verbascum yemense var. yemense (black circles) and V. yemense var. asiricum (black triangles) in the Arabian Peninsula.

##### Notes.

*Verbascum yemense* is a variable species occurring in the southwestern Arabian Peninsula. Al-Hemaid (2001) described *V. asiricum* and *V. chaudharyanum* from the same region based on this variation. However, phylogenetic analyses (Alzahrani et al. 2024) and detailed comparisons of their type specimens indicate that the three names represent the same taxonomic entity. All share glabrescent or sparsely stellate hairs above and dense stellate hairs below, paniculiform inflorescences with single flowers or clusters of 2–4, yellowish filament hairs, and an ellipsoid-ovoid capsule with sparse stellate hairs. Thus, *V. chaudharyanum* is treated as a synonym of *V. yemense*, and *V. asiricum* as a variety.

#### ﻿17b. Verbascum yemense var. asiricum

Taxon classificationPlantaeLamialesScrophulariaceae

(Hemaid) A.Alzahrani stat. nov.

7B52CA0F-46E7-5B27-ADF3-DC61C494BA1F

urn:lsid:ipni.org:names:77374376-1

Fig. 32

Verbascum asiricum Hemaid, Pakistan J. Bot. 33(4): 316 (2001).

##### Type.

Saudi Arabia, 70 km, S.E. of Abha, Abha, 14 March 1980, *I.S. Collenette 2091* (holotype K).

##### Description.

*Pedicel* up to 15 mm long.

##### Distribution in the Arabian Peninsula.

It is an endemic variety to Saudi Arabia, which is known from Abha (Dalagan areas, near Souk Al-Ithnayn, and near Tamniah village), southwestern Saudi Arabia (Fig. 33).

##### Etymology.

The name refers to Asir, the local Arabic name of the Asir Mountains, Saudi Arabia.

##### Specimens examined.

**Saudi Arabia.** • **Abha**: Wadi Dalagan, 27 March 1980, *A. Nader 237* (K); 7 km SE Abha, 14 March 1980, *A. Nader 214* (K); Dalagan road, 12 km SE of Abha, 15 April 1995, *I.S. Collenette 9347* (E [E00095077], K); 70 km, S.E. of Abha, 14 March 1980, *I.S. Collenette 2091* (K); near Souk Al-Ithnayn, head of Wadi Al-Soudah, 50 km S of Abha, 21 August 1983, *I.S. Collenette 4478* (E [E00066944]); Dalagan national park, 30 km SE of Abha, 06 March 1981, *D. Hilesat 118* (BM); Dalagan national park, 30 km SE of Abha, 01 March 1981, *D. Hilesat 59* (BM); near Tamniah village, 19 March 2021, *A. Alzahrani 175* (MUZ).

##### Notes.

This variety is distinguished from var. yemense by its longer pedicels, which reach up to 15 mm, and by its restricted distribution in the Dalagan areas, near Souk Al-Ithnayn, and near Tamniah village in the Abha province of southwestern Saudi Arabia.

## Supplementary Material

XML Treatment for Verbascum

XML Treatment for Celsia

XML Treatment for Staurophragma

XML Treatment for Rhabdotosperma

XML Treatment for Verbascum akdarense

XML Treatment for Verbascum bottae

XML Treatment for Verbascum decaisneanum

XML Treatment for Verbascum deserticola

XML Treatment for Verbascum deserticola var. deserticola

XML Treatment for Verbascum deserticola var. sheilae

XML Treatment for Verbascum eremobium

XML Treatment for Verbascum longibracteatum

XML Treatment for Verbascum medinecum

XML Treatment for Verbascum melhanense

XML Treatment for Verbascum omanense

XML Treatment for Verbascum sarawaticum

XML Treatment for Verbascum saudiarabicum

XML Treatment for Verbascum schimperianum

XML Treatment for Verbascum shiqricum

XML Treatment for Verbascum sinaiticum

XML Treatment for Verbascum transjordanicum

XML Treatment for Verbascum virgatum

XML Treatment for Verbascum yemense

XML Treatment for Verbascum yemense var. yemense

XML Treatment for Verbascum yemense var. asiricum

## ﻿Additional information

### Use of AI

No use of AI was reported.

### Author ORCIDs

Ali Mohammed Alzahrani https://orcid.org/0000-0003-0945-0537

Joana Magos Brehm https://orcid.org/0000-0002-6444-6488

Shahina A. Ghazanfar https://orcid.org/0000-0002-0482-2023

Nigel Maxted https://orcid.org/0000-0002-2112-0947

### Data availability

All of the data that support the findings of this study are available in the main text.
